# Evolutionary conservation of RNA sequence and structure

**DOI:** 10.1002/wrna.1649

**Published:** 2021-03-22

**Authors:** Elena Rivas

**Affiliations:** ^1^ Department of Molecular and Cellular Biology Harvard University Cambridge Massachusetts USA

**Keywords:** conserved RNA structure, covariation, RNA folding

## Abstract

An RNA structure prediction from a single‐sequence RNA folding program is not evidence for an RNA whose structure is important for function. Random sequences have plausible and complex predicted structures not easily distinguishable from those of structural RNAs. How to tell when an RNA has a conserved structure is a question that requires looking at the evolutionary signature left by the conserved RNA. This question is important not just for long noncoding RNAs which usually lack an identified function, but also for RNA binding protein motifs which can be single stranded RNAs or structures. Here we review recent advances using sequence and structural analysis to determine when RNA structure is conserved or not. Although covariation measures assess structural RNA conservation, one must distinguish covariation due to RNA structure from covariation due to independent phylogenetic substitutions. We review a statistical test to measure false positives expected under the null hypothesis of phylogenetic covariation alone (specificity). We also review a complementary test that measures power, that is, expected covariation derived from sequence variation alone (sensitivity). Power in the absence of covariation signals the absence of a conserved RNA structure. We analyze artifacts that falsely identify conserved RNA structure such as the misuse of programs that do not assess significance, the use of inappropriate statistics confounded by signals other than covariation, or misalignments that induce spurious covariation. Among artifacts that obscure the signal of a conserved RNA structure, we discuss the inclusion of pseudogenes in alignments which increase power but destroy covariation.

This article is categorized under:

RNA Structure and Dynamics > RNA Structure, Dynamics and Chemistry
RNA Evolution and Genomics > Computational Analyses of RNA
RNA Evolution and Genomics > RNA and Ribonucleoprotein Evolution

## Graphical abstract

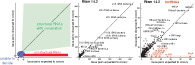

Covariation together with covariation power allow to distinguish between three different conservation patterns in RNA alignments. Structural RNAs with evolutionary signal for a conserved structure have covariation and power (green). Non structural RNAs have power but no covariation (red). RNAs that lack both covariation and power are too conserved in sequence to be able to decide whether they have a conserved structure or not (blue). We show the covariation versus power observed in structural RNAs from the database Rfam (black), and the covariation and power for the long non‐coding RNAs HOTAIR, RepA‐Xist, and ncSRA that do not support a conserved RNA structure (brown)

## 1 INTRODUCTION

Many functional RNAs adopt conserved structures. Conserved structural RNAs are involved in many cellular functions from translation (ribosomal RNA, transfer RNA), to RNA cleavage (RNaseP, ribozymes), protein localization (SRP), or gene regulation at both transcriptional and translational levels (riboswitches, microRNAs). RNA‐Seq probing experiments have revealed numerous expressed and uncharacterized RNAs for which it seems imperative to interrogate whether they perform any relevant function by means of an RNA structure. Here we address the question of how to identify new evolutionarily conserved RNA structures. We will show that a plausible computational prediction by a standard single‐sequence folding method is not evidence of a conserved RNA structure, nor is a computational prediction complemented with chemical probing data. In addition to computational predictions and chemical probing, another standard technique for analyzing an uncharacterized RNA is to compare similar sequences in related organisms. A conserved RNA sequence does not necessarily imply a conserved RNA structure. We will show that it requires a particular pattern of variation to support a conserved RNA structure. We will also show that there are variation patterns that support the absence of a conserved structure as well.

## 2 ALL RNAs, EVEN RANDOM ONES, HAVE PLAUSIBLE STRUCTURES

When presented with a structural RNA, one important statistical question is *to predict its structure*. Information about the structure can bring important clues to decipher the RNA function. Computational methods to predict a secondary structure from the sequence of a structural RNA have a long history (Nussinov et al., 1978; Zuker & Sankoff, 1984). Methods such as Mfold (Zuker, 1989), ViennaRNA (Gruber et al., 2015), RNAstructure (Reuter & Mathews, 2010), NUPACK (Dirks & Pierce, 2003), Sfold (Ding et al., 2004), or GTfold (Swenson et al., 2012) use thermodynamic parameters to predict the most stable folds. Other methods use probabilistic context‐free grammars (Dowell & Eddy, 2004; Rivas et al., 2012), or conditional random fields such as CONTRAfold (Do et al., 2006) or RNAsoft (Andronescu et al., 2003), to produce the most probable set of compatible base pairs given a single RNA sequence. These methods whether they measure structures by free‐energy stability, probability, or an arbitrary score they have the same design principles and use similar algorithms (Rivas, 2013). They search for an optimal final configuration of the whole RNA, and do not take into account the kinetics of the folding process, although there are specific methods that systematically study RNA folding kinetics both computationally (Flamm et al., 2000; Mironov & Lebedev, 1993; Wolfinger et al., 2004) and experimentally (Incarnato et al., 2017). They model base pairs and stacked interactions of base pairs as well as details of the different loops of unpaired nucleotides that appear in an RNA secondary structure. They use dynamic programming algorithms that can calculate the optimal structure or the maximum expected accuracy structure which tends to be more accurate than the former regardless of the model parameterization (Hamada et al., 2009; Lu et al., 2009). For an RNA sequence of length *L*, the algorithms have a complexity of $O\left(L^{3}\right)$ when pseudoknots are not included, and much higher ($O\left(L^{6}\right)$) when pseudoknots are allowed by the model (Rivas & Eddy, 1999).

**FIGURE 1:**
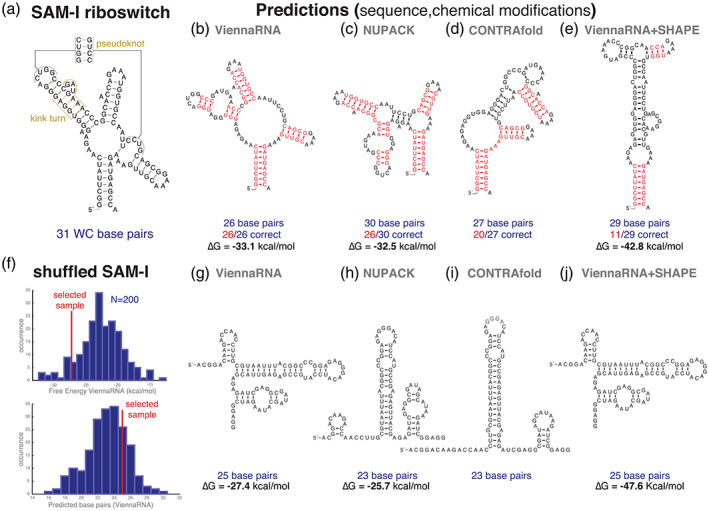
RNA structure prediction from a single sequence with and without chemical modification data. (a) Sequence and structure of a SAM‐I riboswitch from *Thermoanaerobacter tengcongesis* determined by X‐ray crystallography (Montange & Batey, 2006). Watson–Crick base pairs are depicted with a black line. The SAM‐I structure includes a pseudoknot and a kink‐turn motif with three non‐Watson–Crick A‐G pairs. (b–d) Secondary structure predictions for the SAM‐I riboswitch by the software ViennaRNA (program: RNAfold) (Gruber et al., 2015), NUPACK (program: mfe) (Dirks & Pierce, 2003), and CONTRAfold (Do et al., 2006). Base pairs correctly predicted are depicted in red. These prediction methods cannot find pseudoknots or non‐Watson–Crick base pairs. (e) Structure prediction using both the sequence and per residue chemical reactivities obtained using the SHAPE‐Seq 2.0 method, an deposited in the URL (https://rmdb.stanford.edu/detail/SAMRSW_1M7_0001) (Loughrey et al., 2014), predicted using ViennaRNA (RNAfold –‐shape <reactivities> < sequence>). (f) Distributions of the free energies and number of base pairs observed in the best structures (calculated using ViennaRNA) for 200 randomized versions of the *T. tengcongesis* SAM‐I sequence. (g–i) Predicted structures for one shuffled SAM‐I sequence selected for having similar number of base pairs as the ViennaRNA prediction for the real SAM‐I sequence. (j) Structure predicted by ViennaRNA using the same randomized SAM‐I sequence and a set of randomized SHAPE reactivities

The success of these programs in recapitulating the structure of known structural RNAs from a single sequence alone is on average between 65% and 70%, both for the fraction of true base pairs correctly predicted (sensitivity) and the fraction of predicted base pairs that are correct (positive predictive value) (Andronescu et al., 2007; Rivas et al., 2012). This overall average accuracy is comparable for all methods, but there is high variability from sequence to sequence for a given method, and from method to method for a given sequence. These are modest but nevertheless informative results when it is known (or safe to presume) that the sequence is a structural RNA, but whose actual structure is unknown. As an example, Figure 1a shows the known structure of the SAM‐I riboswitch, a bacterial RNA, which includes 31 Watson–Crick (WC) canonical base pairs, and 3 more A:G noncanonical base pairs forming a kink turn RNA motif. In Figure 1b–d, we show different structures predicted by different methods using the *Thermoanaerobacter tengcongesis* SAM‐I riboswitch sequence. ViennaRNA and RNAstructure (not shown in Figure 1) predict a structure with 26 base pairs, all of them correct. The methods NUPACK and CONTRAfold give worse predictions for this sequence. On a different sequence, the situation may be reversed and ViennaRNA could be worse than NUPACK or CONTRAfold. Single sequence RNA prediction methods are generally insufficient to accurately predict base pairs of a structural RNA, specially for large RNAs such as the small and large subunits of rRNA (Doshi et al., 2004).

That is not to say that the secondary structure methods are bad at determining the right structure, rather the problem is inherently not constrained enough by base pairing. That is, there are many different ways to create different plausible structures from the same sequence, with similar number of base pairs and free energy, and it is hard to know which one is correct. To illustrate that point, a straightforward calculation shows that in the *T. tengcongesis* SAM‐I riboswitch sequence, there are 32 helices with at least 3 consecutive base pairs, of which only 5 of them appear in the actual structure. These possible 32 helices cannot occur all at the same time, but using subsets of those 32 helices, one can construct many different consistent structures different from the real one and with comparable stabilities. Some of those structures are the ones identified by the different computational methods in Figure 1b–d. This under‐determination problem increases exponentially with the length of the RNA. For instance, for the human telomerase RNA with 451 nts, the number of possible helixes with at least 3 base pairs is 1716. This “exceedingly large number of potential helices” (Gutell, 2014) is a reason why it is unlikely to predict an RNA structure correctly only from maximizing G:C, A:U, and G:U base pairs. In addition, it has been shown that small perturbation in the thermodynamic parameters, perturbations within the parameters' determination error, can result in important changes in the predicted structure (Layton & Bundschuh, 2005).

If you are by now unsatisfied with this scenario, things get even more interesting, because in most cases, we are presented with RNA transcripts for which we do not even know if they are structured or not.

Given an uncharacterized RNA transcript, the statistical question is not finding a structure, but rather *whether the RNA has a biologically relevant structure or not*. This question requires a different approach. Addressing this question requires evaluating whether we can distinguish folds of real structural RNAs from those of nonstructured RNA sequences. At minimum, we would expect within this framework (that ignores 3D fold considerations) to be able to distinguish between real RNA folds and predicted foldings of random sequences.

Unfortunately, the situation is that in most cases we cannot. Even random sequences have plausible folds, often with similar stability, number of base pairs, and structural complexity as the structures of real RNAs. Thus, predicted structures of random sequences are often effectively indistinguishable from those of real RNAs. As an example, for the SAM‐I riboswitch in Figure 1a, randomized versions of that sequence are predicted to adopt stable folds with a comparable number of base pairs as the original one. In Figure 1f, 22% (45/200) of the random sequences can form at least as many base pairs as the best prediction for the real sequence with 26 base pairs. The median number of base pairs is 24, and no sequence has fewer of 16 predicted base pairs, which still look to the eye like reasonably structured RNAs. The free energy of the real SAM‐I riboswitch sequence (−33.1 kcal/mol for the ViennaRNA best prediction) is distinguishable from shuffled SAM‐I riboswitch sequences as it falls at the edge of the free energy distribution in Figure 1f. The difference is marginal though, and for most RNAs there is not enough signal in the free energy to reliably distinguish real structural RNAs from random sequences (Rivas & Eddy, 2000).

An example of a predicted structure of a shuffled SAM‐I sequence with a free energy of −27.4 kcal/mol and 25 base pairs fold using ViennaRNA is shown in Figure 1g. Other methods produce different folds (Figure 1h–i) with a comparable number of base pairs as the fold of the real sequence. It would be impossible to infer based on these folds whether the shuffled sequence is a real structural RNA or not.

The number of possible helices with at least three base pairs for a random sequence of the length (94 nts) and the base composition (A = 31%, C = 22%, G = 32%, U = 15%) of the SAM‐I riboswitch is 29 ± 12 helices. For a randomized sample of the human telomerase RNA, the total number of potential helices with at least three base pairs is 1572 ± 150. These numbers are not just large, they are comparable to those of the real sequences (32 and 1716 respectively). RNA structure prediction from a single sequence cannot reliably distinguish real structural RNAs from unstructured RNAs or even random sequences.

An additional source of information typically used to predict the structure of an RNA comes from experimentally determined reactivities per‐residue of the RNA molecule when subjected to some chemical modification. For instance, SHAPE‐Seq performs selective 2‐hydroxyl acylation analyzed by primer extension (Loughrey et al., 2014). Most chemical modification methods do not provide information on specific base pairs per se, but reactivities for individual residues. Reactivities are affected by whether the residue is base paired or not, but they do not specify which base it is paired to, except for methods such as PAIR‐MaP (Mustoe et al., 2019). Thus, reactivities do not infer an RNA structure directly. Different approaches have been proposed to incorporate chemical probing data into RNA folding algorithms (Deigan et al., 2009; Eddy, 2014; Sükösd et al., 2013; Washietl et al., 2012; Zarringhalam et al., 2012), which have been adopted into all major single‐sequence RNA structure prediction methods.

Chemical‐probing‐directed RNA structure prediction can help with our first statistical question and result in better structural predictions for bona fide structural RNAs (Sükösd et al., 2013). However, RNA chemical probing by SHAPE or other probing methods do not provide an “experimentally determined structure,” as structures determined using chemical modifications data are sometimes referred to (Somarowthu et al., 2015). Eddy (2014) quantified that there is a 5:1 ratio for a residue with low SHAPE reactivity to be base paired versus unpaired. Figure 1e shows the predicted structure for the SAM‐I riboswitch informed by SHAPE‐seq 2.0 data using the chemical reagent 1m7 obtained from Loughrey et al. (2014). In this particular example, the SHAPE‐informed predicted structure has lower accuracy than the one without it. The situation could be different for other RNAs, and chemical probing data tend to improve single‐sequence RNA structure prediction when a structure can be presumed to exist (Sükösd et al., 2013).

Unfortunately, it has become common practice to accept a structural prediction informed by chemical probing as proof of the existence of RNA structure (see e.g., Watts et al., 2009). However, chemical probing cannot distinguish functional RNAs (with biologically relevant structures) from nonfunctional ones because all RNAs in solution will adopt some folding, and thus will have a chemical probing signature—even random RNA sequences. In Figure 1j, we show a structure prediction for a shuffled SAM‐I riboswitch sequence in combination with a random assignment of the SHAPE reactivities for the real sequence resulting in an apparent RNA structure which one would not be able to reject in comparison to any of the predictions for the real SAM‐I riboswitch structure. RNA structure prediction from single sequence even when informed by experimental chemical probing data cannot address the question of whether an RNA has a biologically relevant structure or not. Instead, we need to turn to evolutionary conservation to approach that question.

## 3 CONSERVED STRUCTURAL RNAs HAVE A DISTINCT PATTERN OF VARIATION

Any functional structural RNA is expected to have some level of structural conservation. The RNA would have to interact with other molecular elements, mostly proteins with conserved structures, and changes that could break the RNA structure would not be easily tolerated if the rest of the machinery is conserved.

Structural conservation is not necessarily synonymous with conservation of the RNA sequence, but some level of sequence conservation is also expected. Homologous structural RNAs can be very divergent, but still have a rate of substitutions at base pairs slower than (and distinguishable from) unconstrained substitutions at the two positions. This effect is better observed when the comparison is restricted to a clade. The slower rate of substitutions at base pairs results from most mutations being rejected, except for double compensatory substitutions that produce 3D functionally equivalent molecules.

**FIGURE 2:**
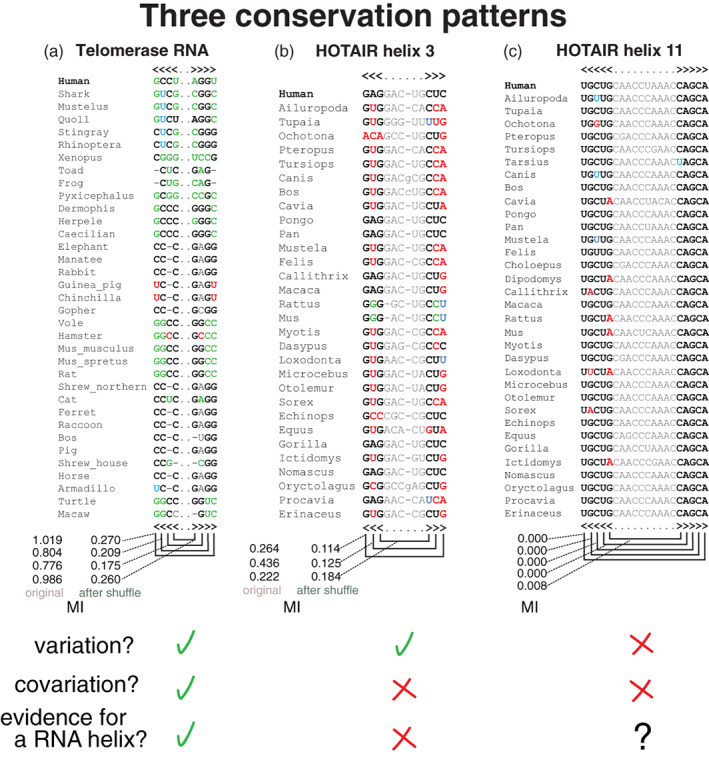
Three different patterns of sequence conservation with different implications for inferring RNA structure. (a) For the vertebrate telomerase RNA, a helix from the Rfam seed alignment (RF00024). The pattern of substitutions (calculated relative to consensus CCCC…GGGG) supports the helix being conserved throughout evolution. (b) From HOTAIR domain 1, putative helix 3 from the structural alignment provided by Somarowthu et al. (2015). The substitutions are mostly incompatible with the annotated helix. (c) Putative helix 11 from the same HOTAIR structural alignment in (b). The small number of changes makes it inconclusive whether the helix exists or not. In green, residues that preserve the structural annotation by making a compensatory base pair substitution relative to the consensus base pair; in blue, a half change (such as G:C to G:U) that also preserves the base pair; in red, changes that break the proposed base pair; and in gray, residues that are not analyzed. We display the mutual information (MI) of each of the original base pairs, and after the residues in each column are permuted to destroy all covariation

For a conserved RNA sequence, the pattern of substitutions observed in an alignment is informative of whether there is a conserved RNA structure or not. Figure 2 shows three different patterns resulting in three different outcomes. In Figure 2a using a helix from the vertebrate telomerase RNA as an example, we observe a pattern of changes in which substitutions tend to preserve A:U, G:C, or G:U base pairs. This pattern indicates a pressure to maintain the helix through evolution. In contrast, the pattern observed in Figure 2b using a putative helix for the RNA HOTAIR (helix 3) indicates that while there is a similar number of mutations as in the telomerase RNA helix, most of those changes appear to be uncorrelated and do not seem to preserve the base pairs annotated in the human HOTAIR sequence as inferred by structural prediction informed by chemical probing data (Somarowthu et al., 2015). Figure 2c shows a different pattern observed in another HOTAIR proposed helix (helix 11). In this case, the lack of variability in the sequence makes it impossible to determine whether the helix is conserved or just one of the many possible helices one can find in any RNA sequence.

### 3.1 Covariation is used to measure structural RNA conservation

Conservation patterns like the one presented in Figure 2a have been long recognized in structural RNAs, and they have been successfully exploited to predict the structure of ribosomal RNA and other RNAs to great accuracy (Gutell et al., 1985; Holley et al., 1965; Michel et al., 1982, 2000; Noller et al., 1981; Pace et al., 1989; Williams & Bartel, 1996). Since the early covariation analyses, computational methods based on mutual information (MI) and other covariation methods have been developed (Akmaev et al., 2000; Gutell et al., 1992).

Different covariation measures can be used to quantify the sequence variation observed in Figure 2a. MI values (Shannon, 1948) such as those reported in Figure 2 help distinguish the variation due to the RNA structure from random changes or no change at all. MI has been used extensively to measure RNA covariation (Akmaev et al., 2000; Dutheil, 2012; Gutell et al., 1994; Yeang & Haussler, 2007). There are many other similar covariation statistics that have been tested for the purpose of identifying conserved RNA structures (Lindgreen et al., 2006). In Rivas et al. (2017), we reported that the G‐test covariation measure (Woolf, 1957) has the best sensitivity and positive predictive value among several different covariation measures tested. The G‐test value for two positions *i*, *j* is defined as

$$G‐\text{test}\left(i,j\right)=2\;\sum_{a,b}{\mathrm{Obs}}_{\mathrm{ij}}^{\mathrm{ab}}\;\log\frac{{\mathrm{Obs}}_{\mathrm{ij}}^{\mathrm{ab}}}{{\mathrm{Obs}}_{i}^{a}\;{\mathrm{Obs}}_{j}^{b}},$$

where *a*, *b* are (non‐gap) residues; ${\mathrm{Obs}}_{\mathrm{ij}}^{\mathrm{ab}}$ is the observed count of *a* : *b* pairs in positions *i*, *j* (only counting when both *a*, *b* are residues), and ${\mathrm{Obs}}_{i}^{a}$ is the observed count of residue *a* in position *i*. G‐test and MI have similar expressions; the G‐test uses observed counts where MI uses residue frequencies. These two differ when the positions have gaps. Two pairs of positions with the same residue frequencies will have the same MI, but the pair with fewer gaps will have higher G‐test value.

It was noticed early on that what constitutes significant covariation evidence depends on the sequence context in which the pairs are embedded (Gutell et al., 1994). To that effect, background corrections have been proposed by subtracting the average covariation of all possible pairs in the alignment from each individual value (Dunn et al., 2007). The average product correction (APC) is the most commonly used background correction. For any covariation measure cov(*i*, *j*), for an alignment of length *L* and 1 ≤ *i*, *j* ≤ *L*, the APC correction is defined as

$$\mathrm{APC}‐\mathrm{cov}\left(i,j\right)=\mathrm{cov}\left(i,j\right)-\frac{\mathrm{cov}\left(i\right)\;\mathrm{cov}\left(j\right)}{\mathrm{cov}},$$

where $\mathrm{cov}\left(i\right)=\frac{1}{L-1}\sum_{j=1\left(j\neq i\right)}^{L}\mathrm{cov}\left(i,j\right)$ and $\mathrm{cov}=\frac{2}{L\left(L-1\right)}\sum_{i=1}^{L-1}\sum_{j=i+1}^{L}\mathrm{cov}\left(i,j\right)$. Most measure of covariation after subtracting a background correction improves their performance for detecting covariation in RNA alignments (Lindgreen et al., 2006). In Rivas et al. (2017), we confirmed those results, and adopted the APC‐corrected G‐test statistic as the default measure of covariation in our analysis program R‐scape (RNA Structural Covariation Above Phylogenetic Expectation).

The next issue is how to use covariation to decide whether an RNA has a conserved structure or not. After all, all pairs of positions in an RNA alignment present some amount of covariation, unless a position is 100% conserved. The question then becomes, when is the covariation score sufficiently large to distinguish a conserved RNA base pair from statistical background and other sources of pairwise covariation signal in RNA sequence alignments?

## 4 DISTINGUISHING RNA COVARIATION FROM INDEPENDENT PHYLOGENETIC SUBSTITUTIONS

Even positions that do not seem to support an RNA base pair have some positive nonzero MI, as illustrated in Figure 2b. Even random sequence alignments show some level of covariation due to statistical noise. What constitutes significant covariation evidence of an RNA structure has to be measured against covariation produced by other sources in the context of the same alignment. Phylogeny is an obvious source of background covariation in any conserved sequence alignment. Covariation methods have tried to correct for this background covariation effect (Dutheil, 2012).

**FIGURE 3:**
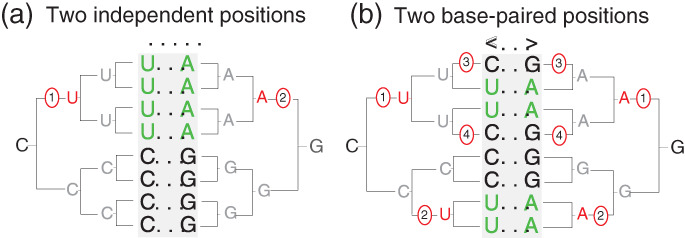
Spurious pairwise covariations can arise from uncorrelated substitutions on a phylogenetic tree. Two aligned positions (gray background) with identical mutual information, (a) one resulting from two independent substitutions (C to U and G to A) that happen to occur at the same branch in the phylogenetic tree, (b) the other resulting from four pairs of compensatory substitutions preserving a canonical RNA base pair (two C:G base pairs becoming U:A, and two U:A becoming C:G). Each of the four pairs of compensatory substitutions occurs at a different branch in the phylogenetic tree

Figure 3 shows an example of two toy alignments that show similar variation but where only one of the cases is associated with a conserved RNA base pair. In Figure 3a, two independent substitutions produce by chance what appear to be a compensatory double mutation. In Figure 3b, eight mutation coordinated as four compensatory pairs preserving a base pair produce descendent sequences with exactly the same pairwise residue frequencies as in Figure 3a.

In Rivas et al. (2017), we designed a statistical test to distinguish between these two different covariation scenarios, implemented in a program named R‐scape. In the R‐scape test, the covariations in an alignment are compared to the distribution of covariation scores observed in simulated alignments of similar degree of divergence and phylogenetic correlation, but where structural correlation has been removed. The simulated alignments reproduce the same number of substitutions in the same branches as the real alignment. But in the simulated alignments, the substitutions for a given branch do not occur at the same positions as in the real alignment, but at different random positions selected from the pool of positions with the same residue. For instance, for the evolutionary history in Figure 3b, the two depicted columns arise from an evolutionary history of four correlated pairs of mutations occurring in four different branches, at just two positions. In the simulated alignments, those mutations will be replaced as eight independent mutations in the same four branches, but occurring at random positions in the alignment that allow those particular mutations.

Using many simulated alignments, we obtain the distribution of covariation scores for an evolutionary history similar to that of the input alignment, but after removing any positional co‐evolution in the real alignment. The empirical distribution for this null hypothesis of covariation due solely to phylogeny is used to estimate the number of expected false positives due to phylogeny, called the E‐value, per pair of positions. The E‐value estimates the expected number of pairs related by phylogeny alone what would have a score at least as large as the pair in question. The smaller the E‐value, the fewer expected pairs related just by phylogeny that would have a comparable covariation score.

### 4.1 Significant covariation in structural RNAs

**FIGURE 4:**
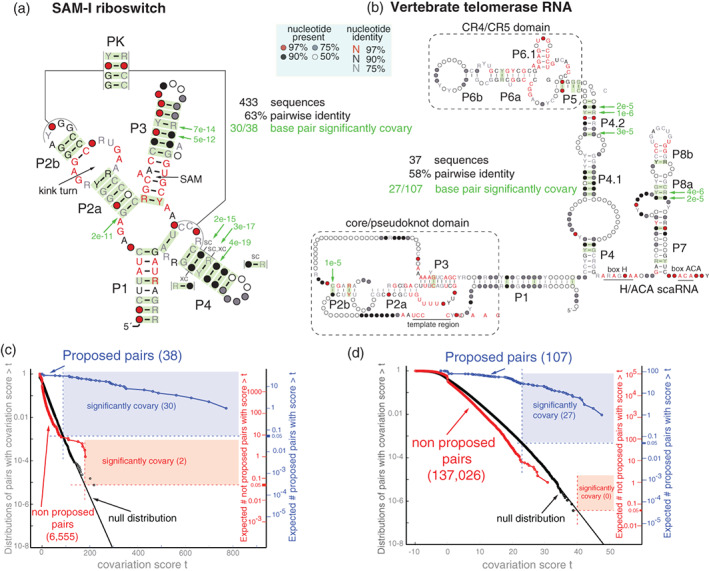
RNA significant covariation above phylogenetic expectation, R‐scape. Statistical test for the structures of the SAM‐I riboswitch and the vertebrate telomerase RNA (vTR) using the Rfam seed alignments (with 433 and 37 sequences respectively). The SAM‐I riboswitch consensus structure is derived from the X‐ray 3D crystal structure (Montange & Batey, 2006). The vTR consensus structure is derived from Zhang et al. (2010). Using R‐scape option ‐s, two independent statistical tests are performed: one for the set of base pairs in the given structures and another for the rest of the pairs (blue and red respectively in panels c and d). (a,b) Depicted in green are the base pairs in the structures that significantly covary with an E‐value <0.05 using R‐scape statistical test for the proposed structures. The top six base pairs with lowest E‐values are marked with an arrow. For the SAM‐I riboswitch structure, 31 of the 38 base pairs covary above the phylogenetic signal. For the vertebrate telomerase RNA, 27 of the 107 base pairs significantly covary. For the SAM‐I riboswitch, there are two significant triple interaction not part of the proposed structure labeled “sc” (side‐covariation) and “xc” (cross covariation) respectively. (c,d) Cumulative distribution of covariation scores for the proposed base pairs (in blue) and all the rest of the pairs (in red). Covariation scores larger than 88 for the base pairs (larger than 179 for the rest of pairs) in the SAM‐I riboswitch are significantly covarying with E‐values <0.05. For the vTR, significant scores are those larger than 23 for the set of base pairs, and larger than 40 for the rest of pairs

Figure 4 shows how R‐scape works using two examples of structural RNAs, the SAM‐I riboswitch with an average length of 110 nts, and the longer vertebrate telomerase RNA (vTR in Figure 4 legend) with 445 nts on average. Figure 4 uses the Rfam seed alignments for these two structural RNAs. Figure 4a,b shows the consensus structures given in the Rfam alignments where base pairs with significant covariation support (E‐values smaller than 0.05) have been marked green. Figure 4a,b shows that in both molecules most helices have covariation support for at least one of the base pairs. Figure 4c,d shows in blue the distribution of covariation scores for the base pairs in the annotated structure. Shaded in blue are the base pairs with E‐value smaller than 0.05, that is those for which their score corresponds to an expected number of false positives smaller than 0.05 under the null distribution (plotted in black). For the SAM‐I riboswitch, this analysis identifies 30 out of the 38 annotated base pairs as significantly covarying, and 27 out of 107 for the vertebrate telomerase RNA.

The R‐scape analysis also calculates the significance of the covariation support for all other possible pairs besides those in the given consensus structure. Figure 4c,d shows in red the distribution of covariation scores for nonbase pairs. In shaded red are those nonbase pairs with a score that would result in an expected number of false positives less than 0.05. For the SAM‐I riboswitch, two tertiary interactions are found significant (named “sc” for side‐covariation and “xc” for cross‐covariation in Figure 4a). They appear to be indirect correlations between residues involved in the highly correlated base pairs of helix P4.

### 4.2 Significant covariation in alternative RNA structures

**FIGURE 5:**
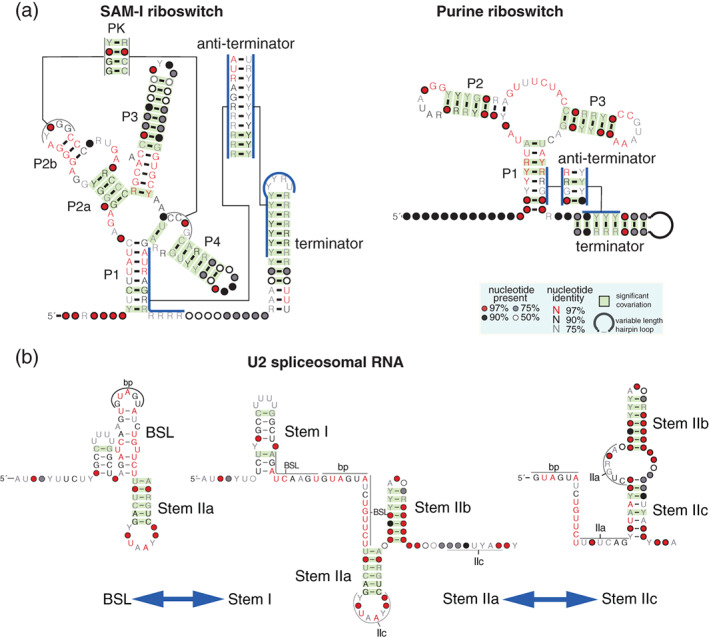
Covariation in alternative RNA structures. (a) R‐scape covariation analysis of the SAM‐I riboswitch and the purine riboswitch from alignments that include both the aptamer and the expression platform sequences, using a consensus structure that includes both the terminator and anti‐terminator alternative and overlapping helices. For the two riboswitches, both the terminator and anti‐terminator helices have covariation support. The SAM‐I riboswitch structural alignment including the terminator sequence was produced by Zhu and Meyer (2015). The purine riboswitch extended structural alignment was obtained from Ritz et al. (2013). (b) Two sets of alternative structures in U2 spliceosomal RNA: The branching interacting stem loop (BSL)/Stem‐I (Perriman & Ares, 2010), and the Stem‐IIa/Stem‐IIc alternative structures (Perriman & Ares, 2007). There is covariation evidence for three of the alternative helices Stem‐I, Stem‐IIa, and Stem‐IIb. The sequences forming the BSL are very conserved and lack covariation. The R‐scape analysis is performed in the Rfam U2 seed alignment (RF00004)

Riboswitches are examples of RNAs with alternative structures with covariation evidence. Figure 4a shows the structure of the SAM‐I riboswitch aptamer, but riboswitches have another functional domain called the expression platform. The aptamer and expression platforms overlap by a region that can form two alternative helices. Figure 5a shows the R‐scape analysis of alignments including both the aptamer and the expression platforms for the SAM‐I riboswitch from Zhu and Meyer (2015) and the purine riboswitch obtained from Ritz et al. (2013). These extended alignments show evidence of covariation in both the terminator and anti‐terminator alternative helices.

Another example of an RNA with conserved alternative structures is the U2 spliceosomal RNA. The Stem‐IIa and Stem‐IIc are two competing structures that promote different splicing steps (Perriman & Ares, 2007). In addition, the branching interacting stem loop (BSL) rearranges with Stem‐I (Perriman & Ares, 2010). Figure 5b shows that there is covariation evidence for the two alternative Stem‐IIa and Stem‐IIb. Stem‐I also has covariation support, but the sequences forming the BSL are very conserved and lack covariation.

### 4.3 Lack of significant covariation in conserved lncRNAs

R‐scape has shown that proposed structures for some long noncoding RNAs (lncRNAs) such as the eutherian HOTAIR (Somarowthu et al., 2015), Xist RNA (Fang et al., 2015; Maenner et al., 2010), and ncSRA (Novikova et al., 2012), although they have been said to be evolutionarily conserved, in fact do not present any statistically significant evidence of structural covariation (Rivas et al., 2017). The published structures proposed for HOTAIR, Xist, and ncSRA were constructed using experimental chemical probing in combination with different prediction algorithms on single sequences (Fang et al., 2015; Maenner et al., 2010; Novikova et al., 2012; Somarowthu et al., 2015), yet alignments of vertebrate homologs with the proposed consensus structure do not show any significant covariation above phylogenetic expectation. This covariation analysis however does not distinguish whether the lack of covariation in these lncRNAs occurs despite sufficient variability as in Figure 2b or whether it is merely due to lack of variability as in Figure 2c. The former case gives evidence against the presence of a conserved RNA structure, while the latter cannot rule out the presence of a conserved structure that could be eventually inferred and supported from a more diverse alignment.

## 5 SEQUENCE VARIATION WITHOUT COVARIATION SIGNALS THE ABSENCE OF CONSERVED RNA STRUCTURE

To distinguish between the two possible scenarios for why covariation support would be absent, Rivas et al. (2020) quantified the covariation potential defined as the number of base pairs expected to covary given the variability observed in the alignment. While the R‐scape E‐values are a measure of false positives expected under the null hypothesis (specificity), power is a measure of the expected true positives (sensitivity). Power is estimated by empirically calculating the probability that bona fide RNA base pairs would be reported as significantly covarying as a function of the variability. For conserved RNA structures, we expect a concordance between covariation and power. Alignments of real structural RNAs often show both covariation and power. Real structural RNAs can produce alignments with low power, for instance when the aligned sequences belong to a specific clade of species. Low power means that there is not conclusive evidence as to whether there is an evolutionarily conserved RNA structure or not. On the other hand, power in the absence of covariation would suggest an evolutionary signal not compatible with RNA structure.

**FIGURE 6:**
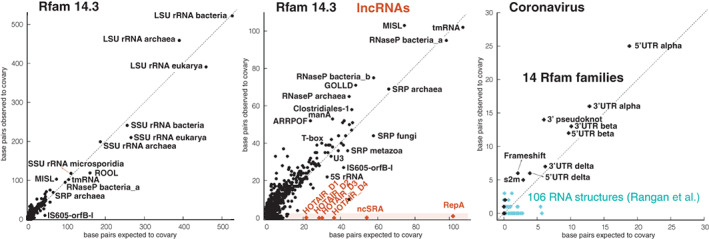
Covariation versus power. Correlation between covariation and power for known structural RNAs, three long noncoding RNAs (lncRNAs) with proposed conserved structures, and structures proposed in coronavirus. (a) Analysis of all 3444 RNA families in Rfam 14.3 seed alignments. (b) Extended scale of the Rfam covariation/power concordance plot showing the analysis of proposed structures of the lncRNAs HOTAIR (Somarowthu et al., 2015), noncoding steroid receptor RNA activator (ncSRA) (Novikova et al., 2012) and RepA (Liu et al., 2017). (c) Concordance plots for 14 coronavirus RNA structures reported by Rfam (violet), and 106 proposed SARS‐CoV‐2 RNA structures Rangan et al. (2020) (cyan). The alignments for the 106 Rangan structures were generated using an Infernal model constructed for each proposed sequence/structure after searching a database of 124 RefSeq Nidovirales genomes (the viral order of which coronavirus is a family) downloaded on May 1, 2020 from NCBI. Alignments are provided in the Supplemental Material

As examples of structural RNAs, Figure 6a shows the covariation versus power for the 3444 RNA families in the Rfam database. There is a strong correspondence between the observed number of covarying base pairs and expected number of base pair to covary given the variability present in the Rfam seed alignments. In contrast, Figure 6b shows that for the lncRNAs HOTAIR, ncSRA, and repA RNA (a fragment of Xist RNA exon 1 that includes the repA repeat) for which conserved RNA structures have been proposed, there is a discordance between the number of expected covariations and the almost complete absence of covarying base pairs (only one found for RepA). This pattern of variation in the absence of covariation is evidence against the presence of a conserved RNA structure in these conserved lncRNAs (Rivas et al., 2020).

### 5.1 Low power means inconclusive evidence for a conserved RNA structure

Another conservation pattern that covariation power helps elucidate is that described in Figure 2c. In that case, low covariation is in concordance with low power, and it is not possible to decide on the presence or not of a conserved RNA structure. This happens when the alignment lacks sufficient variation for covariation analysis.

An example of inconclusive results due to low power is shown in Figure 6c for 106 RNA structures reported in the SARS‐CoV‐2 genome. These RNA structures cover approximately 30% of the SARS‐CoV‐2 genome (Rangan et al., 2020). The structures were obtained by a combination of chemical probing and computational prediction. We built Infernal models (Nawrocki & Eddy, 2013) of each of the 106 RNAs, and searched a collection of 124 Nidovirales from RefSeq, the viral order of SARS‐CoV‐2. We used R‐scape to analyze the covariation and covariation power of the alignments. For comparison, we also analyzed the 14 SARS‐CoV‐2 RNA families in Rfam. Nine of the Rfam coronavirus RNA families have at least five covarying base pairs and similar power. On the other hand, for the majority of the 106 RNA structures in Rangan et al. (2020), it remains inconclusive whether there is a conserved RNA structure (2 RNAs have 3 evolutionarily conserved base pairs, and 10 others have 2). We use the term evolutionarily conserved base pair to describe base pairs significantly covarying according to R‐scape analysis of a particular alignment using an E‐value cutoff of 0.05.

**FIGURE 7:**
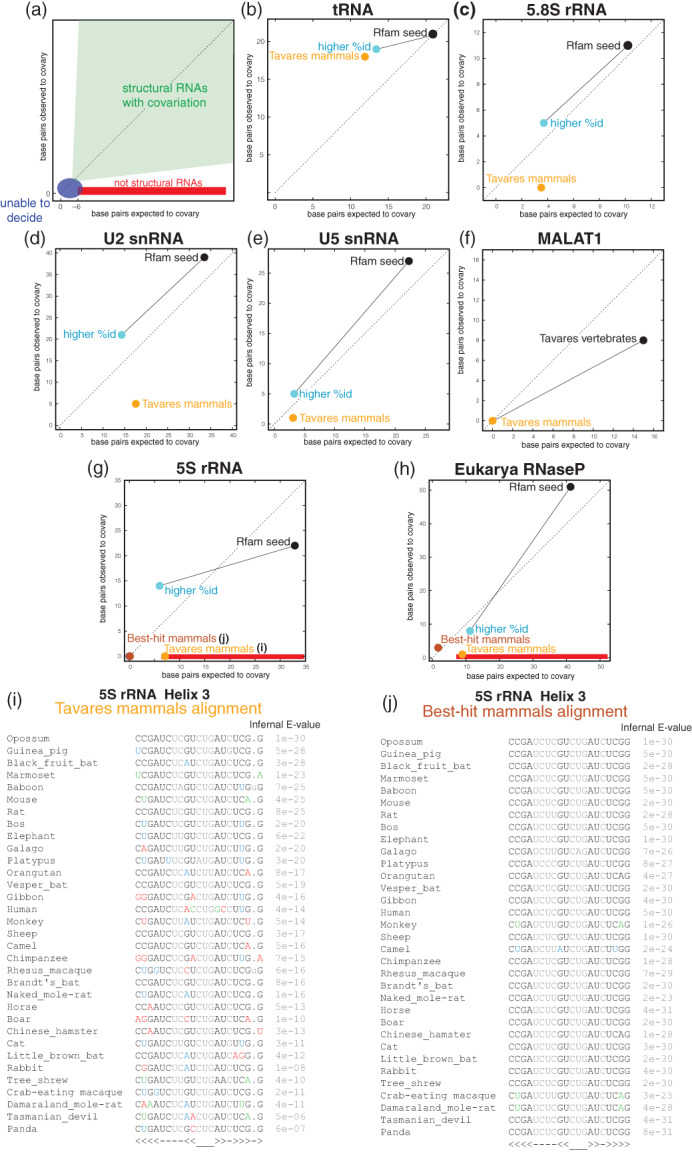
Covariation versus covariation power as a function of sequence diversity. (a) An illustration of the expected covariation and power in alignments of: structural RNAs (green), not structural RNAs (red), RNAs too conserved in sequence to be able to decide whether they have a conserved structure or not (blue). Operationally, the red stripe described the region with at least six base pairs expected to covary, and no covariations observed. We use the term “observed covariation” to describe pairs that are called significant by R‐scape with an E‐value smaller than 0.05. (b–e) For several structural RNAs, the covariation and power for three different alignments are shown: the Rfam seed alignment (black), Tavares et al. (2019) high‐id alignment (blue) derived from the seed alignments by selecting sequences with high percentage identity, and Tavares only‐mammals alignment (orange) produced from the Rfam full sequences using Infernal. (f) For MALAT1, black corresponds to a 132 vertebrates alignment, and orange corresponds to a 13 mammals alignment derived from the previous one, both introduced in Tavares et al. (2019). (g,h) For 5S rRNA and RNaseP RNA, we analyze also another mammals‐only alignment that includes the same species as Tavares but where the selected sequence is the best E‐value Infernal hit per species (maroon). Details of the alignments, their covariation, and power are given in Table 1. (i) Detail of helix 3 of the 5S rRNA Tavares mammals‐only alignment. The human sequence in this alignment is located in chromosome 8 (26,136,880‐26,136,998), and it appears to be a pseudogene. (j) Detail of helix 3 of the 5S rRNA best‐hit Infernal mammals alignment. The human sequence is 1 of 16 identical genes, and belongs to the longest tandem array of 5S rRNA genes located in chromosome 1 (Sørensen et al., 1991) (e.g., chr1:228,632,631‐228,632,749, named RNA5S11). The E‐value of the Infernal search for each species is reported next to the alignment. Human coordinates are from assembly GRCh38/hg38. The alignments are provided in the Supplemental Materials

An analysis presented in Tavares et al. (2019) (reproduced here in Figure 7 and Table 1) observes that alignments of diminishing power result in less covariation. For seven structural RNAs (5S rRNA, 5.8S rRNA, RNaseP, tRNA, U2 snRNA, U5 snRNA, and MALAT1), the Tavares analysis compared the Rfam seed alignments (black in Figure 7) to other restricted alignments obtained by increasing the percentage identity of the sequences (blue), and to alignments obtained by selecting sequences from mammal species only (orange). The observation is that as the power of the alignment decreases, so does the covariation signal. Alignments with less power become increasingly less informative toward assessing the presence or not of a conserved RNA structure. Tavares et al. framed that result as a failure of covariation analysis. The correct interpretation is that those restricted alignments have little variability, and as a result, they do not have sufficient power to decide on whether the structures are conserved or not. Lack of covariation under conditions of no variation does not necessarily imply lack of a conserved structure but the inability to decide on the subject.

**TABLE 1:** Power of covariation and observed covariation in RNA alignments of different diversity

RNA	Alignment	% ID	# seqs	Base pairs	Observed cov	Expected cov	
RNaseP RNA	Rfam seed	48	116	62	51	41 ± 3	Supports structure
	Higher %ID (seed)	69	46	62	8	11 ± 3	Supports structure
	Mammals only (best‐hit)	81	44	55	2	3 ± 2	Cannot assess
	Mammals only (Tavares)	68	45	55	1	9 ± 2	Pseudogenes
5S rRNA	Rfam seed	56	712	34	22	33 ± 1	Supports structure
	Higher %ID (seed)	73	33	34	14	6 ± 2	Supports structure
	Mammals only (best‐hit)	96	33	37	0	0 ± 1	Cannot assess
	Mammals only (Tavares)	75	33	34	0	7 ± 2	Pseudogenes
MALAT1	Vertebrates (Tavares)	71	132	43	8	15 ± 3	Supports structure
	Mammals only (Tavares)	81	13	43	0	1 ± 1	Cannot assess
HOTAIR D1	Tavares	74	37	149	0	22 ± 4	Evidence
HOTAIR D2	Tavares	74	31	134	0	29 ± 4	against
HOTAIR D3	Tavares	68	34	125	0	30 ± 5	a
HOTAIR D4	Tavares	69	31	165	0	37 ± 5	conserved
Rep A	Tavares	71	57	328	1	100 ± 8	structure

*Note:* For different RNAs, we compare the covariation signal in alignments of different power and analyze the relationship between the two. In blue, alignments for which the observed and expected number of covariations are consistent with each other representing either an alignment with enough power to supports a conserved RNA structure or an alignment with little power that cannot provide information about whether a conserved RNA structure exists or not. In red, alignments with power but little or no covariation suggesting the absence of an RNA structure. The alignments' names are color coded as in Figure 7a.

### 5.2 Pseudogenes increase power but destroy covariation

The analysis presented in Tavares et al. (2019) also shows another important point. How erroneously including pseudogenes in an alignment can disrupt a covariation analysis.

Tavares et al. (2019) used some mammal‐restricted alignments to show examples of alignments with variability but not covariation, and concluded that covariation cannot be used to identify structural RNAs (Figure 7g,h). However, their alignments include pseudogenes.

For example, Figure 7i shows the 5S RNA Tavares alignment (helix 3). Tavares' human 5S RNA sequence located in chromosome 8 is a 5S rRNA pseudogene (Sørensen et al., 1991). 5S rRNA is notorious for its large number of pseudogenes, estimated to be over 500 in the human genome (International Human Genome Sequencing Consortium, 2001). Pseudogenes are not subjected to preserving a conserved structure, and their conservation pattern resembles more that of Figure 2b than that of Figure 2a.

A simple fix for avoiding most pseudogenes is to take the best hit per genome (Figures 7j). The best‐hit 5S rRNA mammal alignment with the same species as the Tavares alignment has low power and behaves normally (Figures 7g). This is a cautionary tale; on building alignment for covariation analysis, one should be careful excluding any possible pseudogenes. Pseudogenes can obscure RNA covariation evidence by adding variation inconsistent with an RNA structure.

The alignments for lncRNAs HOTAIR, ncSRA, and repA, on the other hand, do not contain pseudogenes. Thus the discordance found in their alignments with low covariation but high power cannot be attributed (as it is the case for the Tavares mammals‐only alignments) to the presence of pseudogenes in a structural RNA alignment.

## 6 ARTIFACTS THAT FALSELY IDENTIFY CONSERVED RNA STRUCTURE

Other artifacts can have the opposite effect of erroneously creating spurious covariation. We illustrate some of those artifacts using examples from the recent literature.

### 6.1 Misusing the RNA drawing program R2R

**FIGURE 8:**
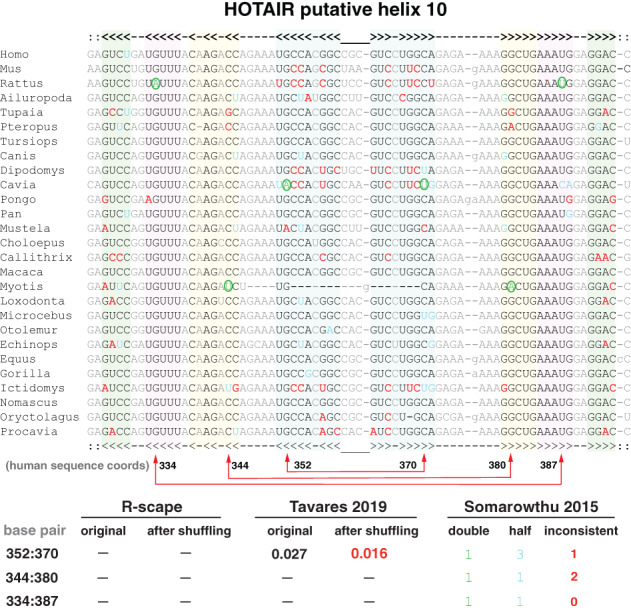
Examples of misidentified conserved RNA base pairs. (a) Example of three base pairs called “significantly covarying” in HOTAIR putative helix 11. The 352:370 pair (in human sequence coordinates) was called significantly covarying in both Somarowthu et al. (2015) and Tavares et al. (2019); the 334:387 and 344:380 pairs were also called significantly covarying in Somarowthu et al. (2015) but not in Tavares et al. (2019). Somarowthu' analysis calls the three base pairs significant solely on the basis that there is one compensatory mutation (circled in green) and less than 10% of the sequences are inconsistent with a canonical base pairs. Tavares' analysis still calls the 352:370 pair significantly covarying even after the residues in each column are permuted to destroy all covariation. Tavares used R‐scape with command: R‐scape ‐‐RAFSp ––window 500 ––slide 100 HOTAIR_D1.sto. Green: compensatory base pair substitutions relative to most abundant canonical base pair; blue: “half flips” (such as GC to GU); red: substitutions inconsistent with proposed base pair. In the current R‐scape, option ‐‐RAFSp can only be used in combination with ‐‐naive to report the full list of RAFS scores without the statistical test of covariation. Derived from Supplementary Figure 4 of Rivas et al. (2017) and Figure 2 of Rivas and Eddy (2018)

The original analysis of the HOTAIR lncRNA alignments reported a structure which the authors say is “evolutionarily conserved” (Somarowthu et al., 2015). For example, three proposed base pairs in HOTAIR helix 10 (Figure 8) were reported by Somarowthu et al. (2015) as covarying.

The Somarowthu et al. (2015) analysis used a data visualization program called R2R that does not perform statistical analysis. The R2R authors explicitly warn against interpreting the R2R drawing for the purpose of covariation analysis (Weinberg & Breaker, 2011). R2R only requires a single compensatory pair substitution to annotate a pair as “covarying,” so long as no more than 10% of the sequences are inconsistent with canonical base pairing of the two positions. Somarowthu et al. (2015) customized R2R's tolerance to allow up to 15% inconsistent base pairs. This is why R2R marked as covarying the three pairs of helix 10 indicated in Figure 8, although the three base pairs include each just one compensatory change, and just as many changes inconsistent with a base pair.

For instance for the 352:370 base pair in helix 10, the 352 position is a highly conserved G, and the 370 position is a mostly conserved C. There is only one pairwise compensatory substitution (an A:U in *Cavia porcellus*), an inconsistent substitution (A:C in *Mustela*), and many inconsistent substitutions at other pairs in the proposed helix. In addition, the mouse/human pairwise comparison in this HOTAIR region shows six substitutions inconsistent with the proposed structure and no compensatory base pair substitutions. Overall, the analysis of the proposed HOTAIR structure is a combination of helices such as proposed helix 10 (Figure 8) and helix 11 (Figure 2c) with a pattern of little variation, mixed with helices such as proposed helix 3 (Figure 2b) showing a pattern of large variation but not covariation.

This HOTAIR analysis shows the danger of not using the appropriate tool to assess covariation. In the words of the R2R authors, as it reads in any R2R output: “R2R is not intended to evaluate evidence for covariation or RNA structure where this is in question. It is not appropriate to use R2R's covariation markings to declare that there is evidence of structural conservation within an alignment. R2R is a drawing program.” A statistical analysis of the same HOTAIR alignments shows that none of the proposed base pairs in Somarowthu et al. (2015) significantly covary (Rivas et al., 2017), while the alignments have sufficient power to find covariation due to RNA structure if those were present (Rivas et al., 2020).

### 6.2 The choice of covariation statistics matters

Different statistics have been developed for analyzing pairwise covariation in RNA alignments. For the purpose of predicting a consensus RNA structure for an RNA *known to have one*, it is advantageous to consider not only covariation but also consistency with base pairing. A completely conserved G column and a completely conserved C column show no covariation, but are consistent with a conserved G:C base pair. A structure prediction program might want to choose a statistics that rewards consistency, though perhaps not as much as covariation. One such statistic developed for RNA structure prediction is RAF (RNAalifold measure) used by the program RNAalifold (Hofacker et al., 2002), and the related statistic RAFS (RNAalifold with stacking) (Lindgreen et al., 2006).

However, for the purpose of distinguishing RNAs that have versus do not have an evolutionarily conserved structure, it is important to use a “pure” covariation measure such as MI or the G‐test used by R‐scape. A statistic like RAF (or RAFS) can erroneously assign significant “covariation” support to completely conserved base pairs.

An example of the inappropriate use of the RAFS statistic to provide support for an evolutionarily conserved RNA structure is Tavares et al. (2019). Figure 2c depicts the Tavares alignment of HOTAIR putative helix 11 showing a five base helix. Tavares' analysis reports the three middle base pairs as “significantly covarying” because RAFS sees them as consistently conserved. There is no covariation and no compensatory base pair substitutions at these three pairs, by definition, because the AGC on the right side of the proposed three pairs is invariant.

Figure 8 shows the HOTAIR alignment for putative helix 10. Tavares calls the 352:370 as significantly covarying (not the other two called by their previous Somarowthu et al. (2015) analysis though). A control for whether Tavares' analysis is detecting covariation is to shuffle the alignment by permuting the residues in each individual column. This destroys all covariation while preserving position‐specific sequence conservation. On a permuted HOTAIR alignment, Tavares' analysis still calls helix 10 positions 352:370 “significantly covarying” (E = 0.016). Using Tavares' ‐‐RAFSp ‐‐window 500 ‐‐slide 100 analysis on the complete HOTAIR D1 alignment, similar numbers of “significantly covarying pairs” are detected in permuted alignments (range 28–39, in 10 different shuffles) as in the original alignment (30, at threshold E < 0.05). More details about this analysis are given in Rivas and Eddy (2018).

### 6.3 A good CMfinder score does not mean an evolutionarily conserved structure

**FIGURE 9:**
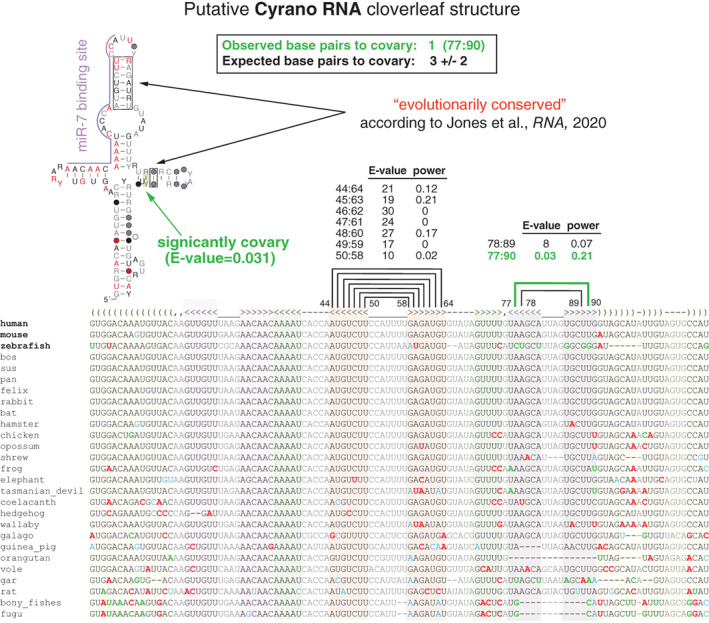
Covariation and power of covariation analysis of the Cyrano RNA putative structure. Proposed cloverleaf structure in the long noncoding RNA Cyrano. Boxed in black, base pairs that Jones et al. (2020) describe as evolutionarily conserved. The alignment was produced by searching 100 vertebrate genomes with an Infernal model built from the human Cyrano RNA cloverleaf sequence and structure provided in Jones et al. (2020). The hypothetical miR‐7 binding site is overlined in purple. The notation describing the alignment positions is given in Figure 4 (blue box)

RNA homology methods such as Infernal (Nawrocki & Eddy, 2013) or CMfinder (Yao et al., 2006) use both sequence and structure conservation, together with consistency with an RNA structure, in order to predict conserved RNA structures. As a result, sequence conservation and/or consistency with an RNA structure can drive a good homology score even in complete absence of covariation. Thus, a good homology score either from Infernal or CMfinder is not guarantee of an evolutionarily conserved RNA structure. Figure 9 shows an example of this situation for the case of a proposed structure for Cyrano RNA (Jones et al., 2020).

Cyrano RNA is a long intergenic noncoding RNA (lincRNA) first identified in zebrafish with homology in vertebrates (Ulitsky et al., 2011). Cyrano RNA includes a putative binding site for microRNA miR‐7. This interaction has been studied for the possible influence of Cyrano on animal behavior (Bitetti et al., 2018; Kleaveland et al., 2018). However, proposed functions of Cyrano RNA have been called into question by recent publications that find that Cyrano is dispensable in zebrafish development (Goudarzi et al., 2019), and dispensable as well for pluripotency of human stem cells (Hunkler et al., 2020). In a recent manuscript, Jones et al. (2020) proposed an RNA structure for Cyrano which is said to be evolutionarily conserved from fish to mammals.

Figure 9 shows the cloverleaf structure for human Cyrano proposed by Jones et al. (2020). The structure was identified using CMfinder. The base pairs framed in two black boxes are said to be evolutionarily conserved because they have a good CMfinder score (Jones et al., 2020).

In order to assess whether the proposed Cyrano cloverleaf structure is evolutionarily conserved, we created the vertebrate alignment shown in Figure 9. The alignment was created using an Infernal model for the human Cyrano cloverleaf sequence and structure. This Cyrano Infernal model was used to search the UCSD 100 vertebrate genome database. An alignment of the best hits was created using the Infernal program cmalign. The alignment with 27 sequences includes mammals, marsupials, and fish species.

The R‐scape analysis of this Cyrano alignment shows one covarying base pair, and consistently it expects 3 ± 2 base pairs to covary. This means the alignment has little variability from which it is not possible to assert the presence of a conserved RNA structure. While the analysis of the overall structure clearly concludes that the alignment lacks variability, the analysis of individual base pairs indicates that at least two of them 45:63 and 48:60 have enough power to start questioning whether the variability observed is consistent with an RNA base pairing at all.

The Cyrano sequence is conserved from fish to human, but it is impossible to decide whether the proposed structure is conserved as well. The fact that the Cyrano sequence is conserved is not enough. This Cyrano region is completely duplicated in human (Cyrano: chr15 41300362 41300860+; Duplication: chr1 240759949 240759453−) as well as in mouse (Cyrano: chr2 119601930 119602533+; Duplication: chr3 129505993 129505339−). The fact that the sequence seems to be mostly consistent with the proposed cloverleaf structure is also not enough, as sequences, even random ones, usually conform with consistent structures. The covariation and power analysis is the way to assess whether the RNA structure is conserved or not. The alignment in Figure 9 is at best inconclusive on that regard.

### 6.4 Misalignments can induce spurious covariation

Observing structural covariation strongly relies on having an alignment where homologous base pairs are aligned respecting the structural constraints of the molecule. Conserved RNA structures can have important differences from species to species: sometimes helices have different number of base pairs; even entire helices may be missing in particular groups of species. The alignment of structural RNAs is further complicated by the fact that the pattern of mutations is more often dictated by base pairing correlations than by simple position conservation. Because helices can have variable number of base pairs and compensatory substitutions, alignments built without taking into account the conserved structure can easily misalign residues in helical regions.

**FIGURE 10:**
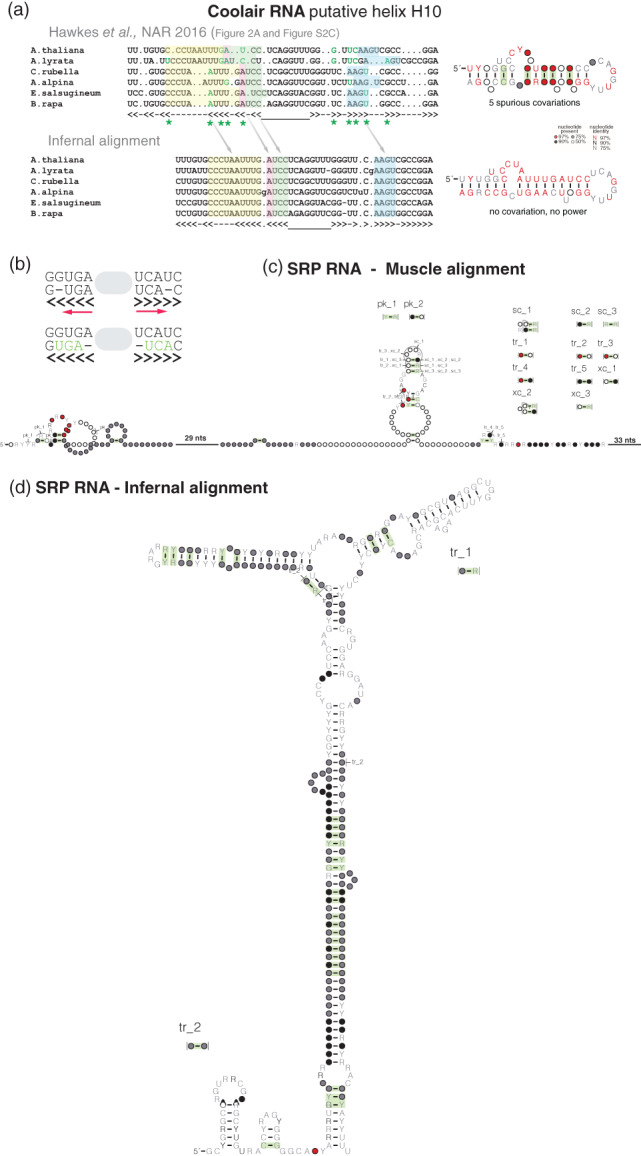
Spurious covariations due to misalignments. (a, top) COOLAIR alignment presented in Hawkes et al. (2016). The green asterisks indicate the position of the five spurious covariations. (a, bottom) Realignment of the same sequences using the program Infernal. The realignment supports the same structure without substitution or gaps in the base paired positions. Derived from Figure 2d of Rivas et al. (2020). (b) Cartoon illustrating the sequence sliding effect that results in spurious covariations. (c) CaCoFold structure prediction for a collection of 312 signal recognition particle (SRP) RNAs from different species including metazoan, protozoa, plants, and bacteria (large and small). The SRP sequences are aligned using the program MUSCLE. (d) Reanalysis of the same sequences, by creating an Infernal model for just one of the sequences (*Zea mays* SRP) using the CaCoFold predicted structure. An alignment is produced using the Infernal program cmsearch with default E‐value cutoff. The Infernal alignment includes 75 out of the 312 SRP sequences that report a significant hit

Perhaps counterintuitively, misalignments can create spurious covariations. Figure 10a shows an example of spurious covariations found in an alignment for the lncRNA COOLAIR (Hawkes et al., 2016). As illustrated in Figure 10b, a misaligned helix in which base paired residues slide relative to the consensus for a fraction of the sequences will result in spurious covariations.

Alignments with base pair misalignments can be identified by performing structural realignments. A technique to identify spurious covariations consists of realigning the sequences using an Infernal model build with the proposed structure. An Infernal realignment of the COOLAIR sequences in Figure 10a shows that the same COOLAIR structure is supported by an alignment that has no variation or covariation. This shows that there is a consistent structure between conserved sequences, but too conserved to infer the presence or not of a conserved RNA structure.

Infernal (Nawrocki & Eddy, 2013) performs structural alignments using both the sequences and a consensus structure. Given a multiple sequence alignment and a consensus secondary structure for all the sequences, Infernal builds a profile context‐free grammar (Durbin et al., 1998) that can be used to find structural homologs (Infernal program *cmsearch*), and to make an alignment of the homologs (program *cmalign*). An Infernal model simultaneously scores conservation, covariation, and consistency with the proposed RNA structure. Using Infernal, the database Rfam has collected reliable alignments of over 3000 structural RNA families (Kalvari et al., 2018).

As a particular case, Infernal can build a model based only in one RNA sequence annotated with a structure. We built an Infernal model for the *Arabidopsis thaliana* sequence and the structure proposed in Figure 10a. The realignment of all sequences using this one sequence/one structure Infernal model produces the COOLAIR alignment in Figure 10b which supports the same structure but the helix is completely conserved.

As a different example, Figure 10c shows for a collection of 312 metazoan, protozoa, plants, and bacteria signal recognition particle (SRP) RNAs, the covariation found in a nonstructural alignment of all sequences created using the method MUSCLE (Edgar, 2004). Most of the covariations are spurious, and they do not relate to the structure of SRP. On the other hand, an alignment of the sequences using the method Infernal by building a one sequence/one structure covariance model results in a very different pattern of covariation that recapitulates the SRP structure (Figure 10d).

## 7 DISTINGUISHING BETWEEN DIRECT AND INDIRECT COVARIATION

Direct coupling analysis (DCA) is a method of describing residue‐to‐residue correlations by inferring the direct pairwise statistical interactions in a biological sequence, as opposed to indirect interactions, where two residues that do not directly interact are correlated because both interact to a third residue resulting in a network effect (Weigt et al., 2009).

DCA methods are based on the statistical mechanical Potts model (Potts, 1952). Potts models are a theoretical advance relative to other correlation measures taken directly from the alignment, such as the above mentioned G‐test and MI which cannot distinguish direct from indirect interactions.

Indirect interactions in RNA can appear due to base triples and other tertiary interactions (Batey et al., 1999). RNA bases have three distinct edges: the WC, Hoogsteen, and Sugar edges. RNA base pairing occurs by direct interaction between any two edges which can form at least two hydrogen bonds, and can have *cis* or *trans* orientation resulting in 12 different types of direct base pairs (Leontis & Westhof, 2001). The most common pairs are *cis* WC base pairs, all other 11 types are usually referred to as the non‐Watson–Crick (non‐WC) base pairs. Triple base pairs form when a base directly interacts with two other bases using two different edges (a found example is an A forming a *cis* WC pair with a U and a Hoogsteen‐type pair with another U). Base triples can induce indirect correlations between the other two bases. Because non‐WC direct interactions tend to show low covariation signal, indirect interactions due to base triples which involve at least one non‐WC pair are not expected to have strong covariation signal.

A Potts model describes the probability of a sequence *s*_1_…*s*_*L*_ as,

(1) $$P\left(s_{1}\ldots s_{L}\right)=\frac{1}{Z_{L}}\exp\left\{+\sum_{i<j}J_{\mathrm{ij}}\left(s_{i},s_{j}\right)+\sum_{i}h_{i}\left(s_{i}\right)\right\}.$$

The parameters {*J*_*ij*_}_*i* < *j*_ are referred to as the *direct couplings*. The partition function *Z*_*L*_ provides the appropriate normalization over all possible sequences of length *L*.

The Potts model in Equation (1) is the maximum entropy probabilistic model (Jaynes, 1982) that reproduces observed arbitrary single and pairwise residue frequencies. Thus, for a Potts model to be consistent with an alignment, it has to satisfy that the marginal probabilities for one and two positions are equal to the corresponding empirical frequencies *f*_*i*_(*a*) and *f*_*ij*_(*a*, *b*) observed in the alignment, that is,

(2) $$\underset{\overset{\wedge }{s_{i}}}{\sum_{s_{1}}\ldots \sum_{s_{L}}}\;P\left(s_{1}\ldots s_{L}\right)=f_{i}\left(s_{i}\right),$$

(3) $$\underset{\overset{\wedge }{s_{i},s_{j}}}{\sum_{s_{1}}\ldots \sum_{s_{L}}}\;P\left(s_{1}\ldots s_{L}\right)=f_{\mathrm{ij}}\left(s_{i},s_{j}\right),$$

where 1 ≤ *s*_*i*_ ≤ *K*, for an alphabet of size *K*.

DCA methods calculate the correlation between two positions *i*, *j* using the direct information (DI) statistic (Weigt et al., 2009),

(4) $$\mathrm{DI}\left(i,j\right)=\sum_{a,b}P_{\mathrm{dir}}^{\mathrm{ij}}\left(a,b\right)\log\frac{P_{\mathrm{dir}}^{\mathrm{ij}}\left(a,b\right)}{f^{i}\left(a\right)f^{j}\left(b\right)},$$

where the “dir” probabilities

(5) $$P_{\mathrm{dir}}^{\mathrm{ij}}\left(a,b\right)\equiv \frac{\exp\left\{+J_{\mathrm{ij}}\left(a,b\right)+{\hat{h}}_{i}\left(a\right)+{\hat{h}}_{j}\left(b\right)\right\}}{\sum_{a^{\prime },b^{\prime }}\exp\left\{+J_{\mathrm{ij}}\left(a^{\prime },b^{\prime }\right)+{\hat{h}}_{i}\left(a^{\prime }\right)+{\hat{h}}_{j}\left(b^{\prime }\right)\right\}}$$

depend on the Potts model coupling parameters *J*_*ij*_(*a*, *b*), and on some modified parameters ${\hat{h}}_{i}\left(a\right),{\hat{h}}_{j}\left(b\right)$ defined by the conditions $\sum_{j}\sum_{b}P_{\mathrm{dir}}^{\mathrm{ij}}\left(a,b\right)=f_{i}\left(a\right)$, and $\sum_{i}\sum_{a}P_{\mathrm{dir}}^{\mathrm{ij}}\left(a,b\right)=f_{j}\left(b\right)$. The DI scores are usually corrected using the APC (Dunn et al., 2007).

DCA methods have proven successful in predicting amino acid contacts from protein alignments, which in turn can be used to predict a protein 3D structure (Ekeberg et al., 2013; Jones et al., 2012; Kamisetty et al., 2013; Marks et al., 2011; Morcos et al., 2011). DCA models have been applied as well to calculate RNA covariations in structural RNA alignments (De Leonardis et al., 2015; Weinreb et al., 2016).

Using DCA statistics requires inferring the coupling parameters from the given alignment. That is not a trivial process, and several different DCA methods exist depending on different optimization methods to train the coupling parameters. For RNA, several methods have been developed using pseudo‐maximum likelihood optimization such as DCA scores (De Leonardis et al., 2015), and evolutionary couplings (ECs) (Weinreb et al., 2016); and others using Boltzmann learning such as BL‐DCA (Cuturello et al., 2020).

Here, we study the effectiveness of these methods in predicting RNA base pairs, and we compare the ECs and BL‐DCA scores to those of a simpler G‐test statistic which does not require parameter training and in principle does not distinguish between direct and indirect interactions.

**FIGURE 11:**
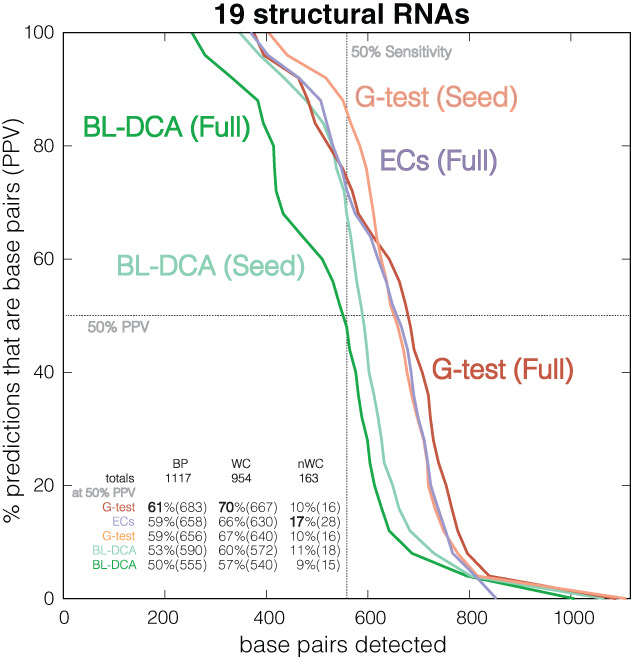
Comparison of different measures of covariation on 19 structural RNAs. We report the number of total base pairs detected as a function of the positive predictive value (PPV, fraction of predictions that are base pairs). G‐test scores are calculated using R‐scape (option ––naive). EC (evolutionary coupling) scores are those provided in Weinreb et al. (2016) on the same alignments. BL‐DCA scores are calculated with the code provided in Cuturello et al. (2020). Dashed lines correspond to 50% PPV (horizontal) and 50% sensitivity (vertical). Results for each structural RNA are given in Figure S1 and Table S1. The seed alignments come from Rfam v14.2. The full alignments are those provided in Weinreb et al. (2016). The annotation of both Watson–Crick (WC) as well as non‐Watson–Crick (non‐WC) base pairs are derived from the PDB files using the program RNAview (Yang et al., 2003) (R‐scape option: ––pdb)

For 19 structural RNAs with experimentally determined 3D structures, we report the number of base pairs detected as a function of the positive predicted value (PPV or fraction of the detected base pairs that are true base pairs) for each of the three methods (Figure 11 and Figure S1). The list includes two ribozymes (RNaseP RNA and GlmS), tRNAs and selenocysteine tRNA, transfer‐messenger RNA (tmRNA), 5.8S rRNA, two SRP RNAs (metazoan and bacterial), group II intron, and 10 riboswitches. For each RNA, we use two different Rfam alignments (seed and full) annotated with the same RNA structure. The annotated base pairs are inferred from crystal structures and include both WC and non‐WC (Table S1).

Overall, our experiments show that for structural RNAs, there is little performance difference using DCA methods versus a simpler G‐test statistic. For most RNAs, we observe a sensitivity threshold at which there is a sharp transition from high PPVs to low PPVs (Figure S1). This sensitivity threshold seems to be mostly dependent on the alignment, and all three methods tend to perform closely to each other (with some variability across RNAs) by the time it is reached. In Table S1, we report the sensitivity threshold, operationally measured at 50% PPV.

Figure 11 summarizes these results. We observe that the sensitivity (measured at 50% PPV) of the G‐test versus DCA methods is almost identical: 61% of base pairs detected using G‐test versus 59% with ECs (Figure 11 and Table 2). This similarity is likely to be the consequence that, unlike proteins, most RNA interactions are direct. The detection of WC base pairs ranges from 70% with G‐test to 66% with ECs. DCA couplings seem to provide a small increase in the determination of non‐WC bases (from 10% sensitivity at 50% PPV with G‐test to 17% with ECs). This result could be because some non‐WC interactions create network effects as they are not necessarily disjoint interactions. On the other hand, the time requirements for training a Potts model are vastly larger than those of calculating the G‐test statistic (Table 2 and Table S1).

**TABLE 2:** Comparison of RNA base pair detection by different covariation measures on a set of 19 structural RNAs

	Base pairs		Rfam alignment	Covariation method	Detected at 50% PPV		Time (min)	
RNA	WC	Non‐WC			WC	Non‐WC	Avg	Max
19 RNAs	954	163	Seed	G‐test	67% (640/954)	10% (16/163)	0.03	0.11
				BL‐DCA	60% (572/954)	11% (18/163)	378.64	1999.27
			Full	G‐test	**70%** (667/954)	10% (16/163)	3.61	33.53
				BL‐DCA	57% (540/954)	9% (15/163)	55.79	279.37
				ECs	66% (630/954)	**17%** (28/163)	—	—

*Note:* We report the total number of detected base pairs (sensitivity) at 50% positive predicted value (PPV, the fraction of the detected base pairs that are true base pairs). G‐test APC corrected scores are calculated using the program R‐scape (option: ‐‐naive). BL‐DCA scores derived from DCA couplings trained using Boltzmann learning are calculated using the code provided with (Cuturello et al., 2020). Evolutionary couplings (ECs) scores calculated from Potts models trained using pseudo‐maximum likelihood optimization come directly from (Weinreb et al., 2016). Running times for ECs were not given.

## 8 OTHER SOURCES OF COVARIATION

A conserved RNA structure results in covariation, but a conserved RNA structure is not the only source of covariation in an RNA alignment. The method R‐scape (Rivas et al., 2017) introduced an empirical model to test the hypothesis of covariation due just to the phylogenetic relationships between the sequences without involving any correlations between specific positions. Using a null model of phylogenetic covariation has enabled identification of significant covariations in alignments of many structural RNAs (Rivas et al., 2020).

**FIGURE 12:**
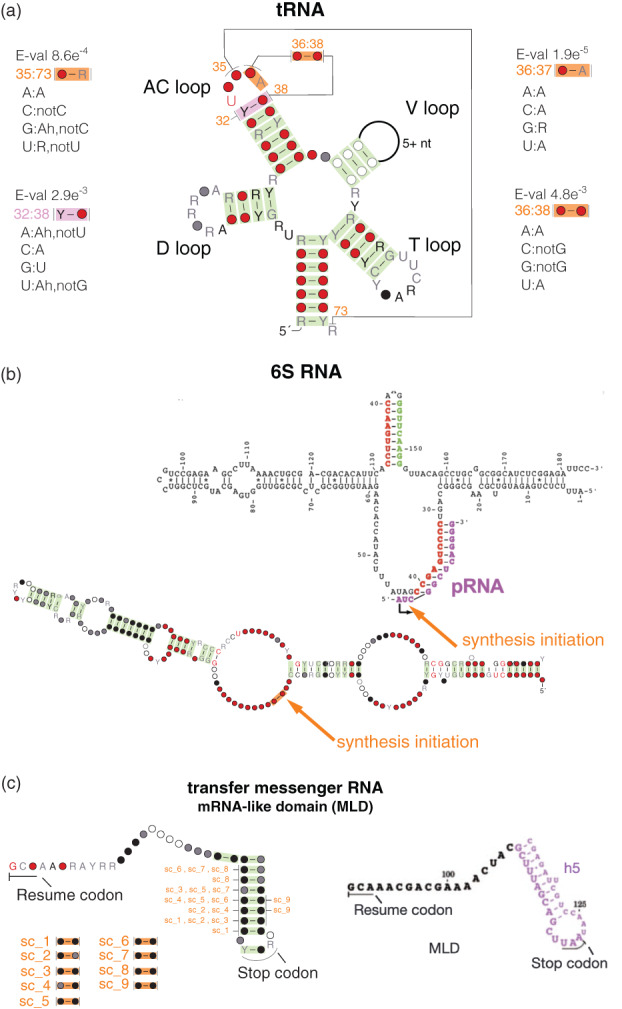
Covariation not related to RNA structure. In orange, significant covariations not attributed to RNA structure. In green, significant covariations attributed to RNA structure. (a) The tRNA alignment shows three significantly covarying pairs in the AC loop not related to the RNA structure. For each pair, we provide information about the observed correlations. A:B mean that probability of finding B at the 3′ end of the pair, given there is an A at the 5′ end, that is *P*(*B*| *A*), is higher than 0.85; A:Bh means that it is between 0.5 and 0.85; and A:notB means that the probability of B given A is lower than 0.05. In pink, we also show a singly hydrogen‐bonded base pair with covariation support found at the junction of the AC loop and stem (Auffinger & Westhof, 1999). Some covarying pairs between the D and T loops also due to RNA structure are omitted for clarity. (b) In 6S RNA, one covarying pair between two residues contiguous in the backbone involving the first position of the RNA product (pRNA) produced by the molecule (Chen et al., 2017). (c) Multiple short‐range covariations in the mRNA‐like domain of the tmRNA (Ramrath et al., 2012). Many of the residues also show significant covariations due to the RNA structure. The covariation analysis was performed in the corresponding Rfam seed alignments using R‐scape and CaCoFold. Figure is derived from Figure 5 and Figure S6 of Rivas et al. (2020)

However, a significant covariation above phylogenetic expectation does not mean that it has to be due to a conserved RNA structure. We have observed various sources of nonphylogenetic and non‐RNA structure covariations while analyzing many RNA alignments (Rivas, 2020). Three examples observed in Rfam seed alignments are given in Figure 12. One source of covariation not due to base pairing are interactions with other molecules. Examples are: (1) in Figure 12a: a covariation between the tRNA middle anticodon position (residue 35) and the discriminator located at the 5′ end of the molecule (residue 73). Both the discriminator and the anticodon bind to the tRNA synthetase, and they are both involved in determining the aminoacylation identity of the tRNA (Giegé et al., 1998). There are two other significant covariations in the AC loop involving the 3′ end position of the anticodon (residue 36) and other AC loop residues. Those covarying pairs are 36:37 and 36:38. The correlations observed in these two pairs appear to be related to translational efficiency (Yarus, 1982). Another AC loop covariation observed between positions 32:38 and adjacent to the AC stem is a single hydrogen‐bond base pairs (Auffinger & Westhof, 1999). (2) In Figure 12b: a covariation in 6S RNA involving two contiguous residues at which the synthesis of the RNA product initiates. The 6S RNA structure mimics an open promoter and serves as a transcription template.

Figure 12c shows another source of covariation not due to RNA structure. The tmRNA is responsible for removing defective mRNAs without a stop codon stacked at the ribosome by providing a short mRNA template ending on a stop codon. For the mRNA‐like domain (MLD) of tmRNA, we observe both covariations associated to RNA structure (part of the MLD sequence is involved in an RNA helix), as well as multiple other covariations between contiguous residues due to the coding structure of the MLD region.

## 9 POSITIVE AND NEGATIVE EVOLUTIONARY INFORMATION TO PREDICT RNA STRUCTURE

The development of power calculations, in addition to significance testing, makes it possible to identify pairs of bases which have plenty of variation, yet not significant covariation. Such pairs of positions are unlikely to form consensus RNA base pairs. We call this “negative” evolutionary information (evidence against a conserved base pair). RNA structure prediction can be made more reliable by using both positive and negative evolutionary information in the form of base pairs that covary and base pairs that vary but do not covary. Given an RNA alignment, the method CaCoFold infers a consensus RNA structure that incorporates all covarying base pairs and avoids all negative base pairs (Rivas, 2020). CaCoFold uses a battery of probabilistic folding grammars that incorporate in layers all observed covarying pairs. A visualization of the resulting structure and the significant covariation that supports it helps identify which parts of the predicted structure are reliable based on their covariation signal, and which are only a prediction consistent with RNA base pairing.

**FIGURE 13:**
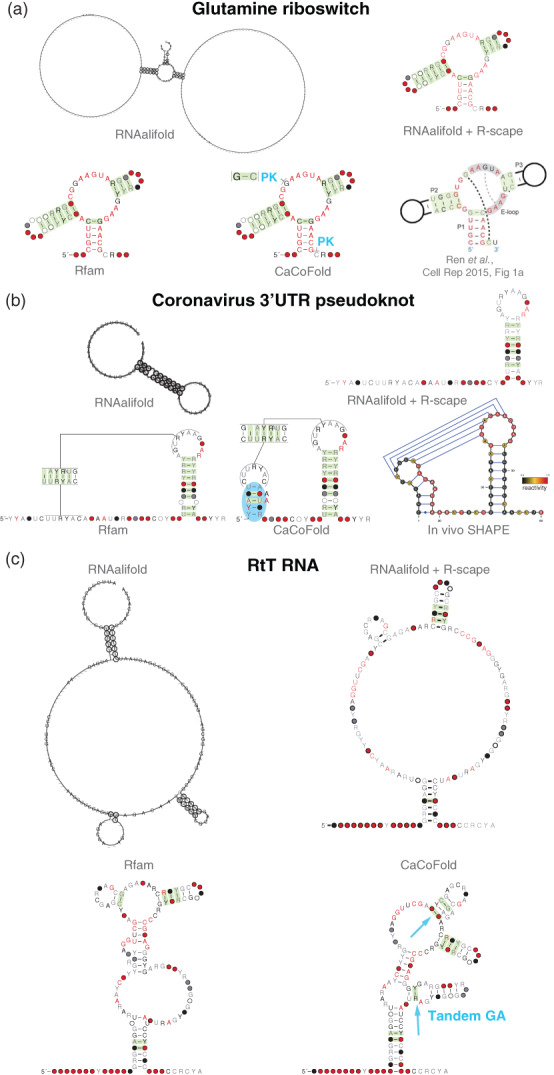
RNA structure prediction using evolutionary information. Comparison of structural predictions by the methods: RNAalifold (Bernhart et al., 2008), R‐scape covariation analysis on the RNAalifold predicted structure (Rivas et al., 2017), Rfam (Kalvari et al., 2018), and CaCoFold (Rivas, 2020). For: (a) the glutamine riboswitch which includes a crystal structure, (b) the coronavirus 3′UTR pseudoknot, which includes results from in vivo SHAPE data (Manfredonia et al., 2020), and (c) the RtR RNA, an RNA element from the tyrT operon of *Escherichia coli*. The structures produced with RNAalifold incorporate the Rfam structure as a constraint (option ––SS_cons). The structures produced with CaCoFold perform the covariation analysis using the R‐scape two‐set mode that tests the Rfam proposed structure (option ‐s ––fold). In blue, motifs identified by covariation alone. Figure is derived from Figures S4, S5, and S7 of Rivas et al. (2020)

Covariation analysis helps identify structural elements that otherwise would remain undetected, such as a single base pair pseudoknot in the glutamine riboswitch (Figure 13a). The pseudoknot is not reported by Rfam or methods like RNAalifold, but because the base pair significantly covaries, R‐scape confirms the interaction, and CaCoFold integrates the base pair as part of the structure. A G:A interaction reported in the E‐loop is very conserved, and no covariation is detected.

Covariation helps predict new structural elements. For instance, for the coronavirus 3′UTR pseudoknot, the covariation analysis of the proposed Rfam structure identifies a new hairpin loop with an AA bulge, a standard RNA motif (Lilley, 1995). The extra proposed helix converts the already identified pseudoknot between a hairpin loop and a single stranded region to a kissing loop pseudoknot between two hairpin loops (Figure 13b). In vivo SHAPE data for SARS‐CoV‐2 provides support only for one of the three helices in this 3′UTR motif, even though all three helices have covariation support (Manfredonia et al., 2020).

Covariation also provides a critical assessment of which parts of the structure are well determined versus which are not. Figure 13c shows predictions for the structure for the vTR RNA (repeat structure of the *Escherichia coli* tyrT operon). Covariation helps identify one additional helix with covariation support which frames a tandem GA, another recurrent RNA motif (Gautheret et al., 1994). It also helps extend another helix due to additional covariation support. We also observe that parts of the structure of the vTR RNA (Figure 4b) are still unconfirmed by the current evolutionary information as there is one proposed helix without any covariation support.

## 10 FINAL REMARKS

A conserved structural RNA implies a slower rate of substitutions at base paired positions, thus conserved structural RNAs from a clade will result in alignments with sequence conservation. However, a conserved RNA sequence does not imply the existence of a conserved RNA structure. Fortunately, there is a distinctive pattern of sequence change associated to a conserved RNA structure different from that of other conservation signals. The identification of novel RNA structures from uncharacterized conserved RNAs is enabled by covariation analysis, and a method to assess which covariations can significantly be excluded from having a phylogenetic source.

Single‐sequence methods of RNA structure analysis (computational and/or experimental) do not reliably identify conserved RNA structure. Covariation and variation information found in alignments of conserved RNAs help distinguish conserved RNA structures from conserved RNAs not supporting a structure. On the other hand, conserved RNA structure cannot be inferred from conserved RNAs without variability.

Covariation analysis requires a covariation measure and a test to distinguish covariation due to RNA structure versus covariation due to other sources. Acceptable covariation statistics are those that measure covariation alone, such as MI, G‐test, or DCA couplings. Covariation measures that also score conservation and consistency with an RNA structure are not acceptable to decide on the presence of a conserved RNA structure as both conservation and consistency occur frequently in RNA sequences, almost with independence of the presence or not of an RNA structure.

R‐scape provides a statistical test to identify base pairs with covariation above phylogenetic expectation. Statistically significant pairwise covariation could still sometimes be due to other sources than a conserved RNA structure. The method CaCoFold which proposed an RNA structure with arbitrary topology using all covariation information present in the alignment is able to display the RNA structure supported by covariation, other covarying interactions not attributed to RNA base pairing, and the regions of the structure with poor determination due to the absence of covariation support.

The accuracy of alignments is fundamental in recovering the evolutionary signals left by a conserved RNA structure. Most alignment algorithms assume a position independent pattern of substitutions which often disrupts the pattern of structural RNAs and may even induce spurious covariation. Starting from a one‐sequence/structure Infernal model to produce the alignment of all conserved sequences is usually better (even if the one‐sequence structure is only partially correct) than using alignments by other standard methods. Emphasis on improving RNA structural alignment methods so that they can be informed not just by the nested base pairs but by all arbitrary RNA base pairing will greatly help the identification of novel conserved structural RNAs.

There are other RNA homology methods comparable to CMfinder that attempt to extract from a genome regions with a conserved RNA structure such as QRNA (Rivas & Eddy, 2001), RNAz (Gruber et al., 2010), or EvoFold (Pedersen et al., 2006). RNA genefinding efforts have been effective in small bacterial genomes (del Val et al., 2007; Rivas et al., 2001), but in eukaryotic genomes they produce too many predictions with too low specificity (Parker et al., 2011; Pedersen et al., 2006; Torarinsson et al., 2006, 2008; Washietl et al., 2005, 2007, 2011). These methods use both sequence and structure conservation. On the other hand, R‐scape measures only covariation and has a tunable false discovery rate, which opens a new line of attack on structural RNA genefinding (Rivas et al., 2017).

## CONFLICT OF INTEREST

The author has declared no conflicts of interest for this article.

## RELATED WIREs ARTICLE

Unraveling the structure and biological functions of RNA triple helices

## Supporting information


**TABLE S1** Detection of RNA base pairs by different covariation measures. The Rfam seed alignments come from Rfam v 14.2. The Rfam full alignments are those provided in Ref. Weinreb et al., 2016. The structural annotation was derived from the PDB files using the program RNAview (Yang et al., 2003), obtained by the program R‐scape using option: –pdb. For a given RNA family, both the Seed and Full alignments have annotated the same structure. The structure includes both the Watson‐Crick (WC) base pairs as well as the non‐canonical base pairs (nonWC). G‐test scores are calculated using the program R‐scape (using option: –naive), and they include the average product correction (APC) (Dunn et al., 2007). Scores from DCA couplings trained using Boltzmann Learning optimization (BL‐DCA) are calculated using the code provided with Ref. Cuturello et al., 2020. ECs scores trained using pseudo‐maximum likelihood optimization come directly from Ref. Weinreb et al., 2016. Running times for ECs are unavailable. PPV stands for positive predictive value, and it is the fraction of the detected basepairs that are true base pairs.

**FIGURE S1** Comparison of different measures of covariation on 19 structural RNAs with experimentally determined structures. For each structural RNA, we compare the performance of three different methods, on two different alignments. One of the methods is G‐test, a simple covariation measure calculated directly from the alignment (generated by the software R‐scape using option ––naive). The other two are DCA methods: EC_RNA (Weinreb et al., 2016) uses pseudo maximum likelihood, and BL‐DCA (Cuturello et al., 2020) uses a Boltzmann Machine to optimize the DCA coupling. All covariation methods use an APC correction (Dunn et al., 2007). The Seed alignments are from Rfam v14.2. The Full alignments are those used in (Weinreb et al., 2016). The base pairs has been inferred from a crystal structure (details given in Supplemental Table S1). The dashed lines correspond to 50% PPV (horizontal) and 50% sensitivity (vertical).

## Data Availability

A tarball with the data used in this manuscript is located at http://rivaslab.org/publications/Rivas21/Rivas21-review_supplement.tar.gz.

## References

[wrna1649-bib-0001] Akmaev, V. R. , Kelley, S. T. , & Stormo, G. D. (2000). Phylogenetically enhanced statistical tools for RNA structure prediction. Bioinformatics, 16, 501–512. 10980147 10.1093/bioinformatics/16.6.501

[wrna1649-bib-0002] Andronescu, M. , Aguirre‐Hernandez, R. , Condon, A. , & Hoos, H. H. (2003). RNAsoft: A suite of RNA secondary structure prediction and design software tools. Nucleic Acids Research, 31, 3416–3422. 12824338 10.1093/nar/gkg612 PMC169018

[wrna1649-bib-0003] Andronescu, M. , Condon, A. , Hoos, H. H. , Mathews, D. H. , & Murphy, K. P. (2007). Efficient parameter estimation for RNA secondary structure prediction. Bioinformatics, 23, 19–28. 10.1093/bioinformatics/btm223 17646296

[wrna1649-bib-0004] Auffinger, P. , & Westhof, E. (1999). Singly and bifurcated hydrogen‐bonded base‐pairs in tRNA anticodon hairpins and ribozymes. Journal of Molecular Biology, 292, 467–483. 10497015 10.1006/jmbi.1999.3080

[wrna1649-bib-0005] Batey, R. T. , Rambo, R. P. , & Doudna, J. A. (1999). Tertiary motifs in RNA structure and folding. Angewandte Chemie (International Ed. in English), 38, 2326–2343. 10458781 10.1002/(sici)1521-3773(19990816)38:16<2326::aid-anie2326>3.0.co;2-3

[wrna1649-bib-0006] Bernhart, S. H. , Hofacker, I. L. , Will, S. , Gruber, A. R. , & Stadler, P. F. (2008). RNAalifold: Improved consensus structure prediction for RNA alignments. BMC Bioinformatics, 9, 474. 19014431 10.1186/1471-2105-9-474 PMC2621365

[wrna1649-bib-0007] Bitetti, A. , Mallory, A. C. , Golini, E. , Carrieri, C. , Gutiérrez, H. C. , Perlas, E. , Pérez‐Rico, Y. A. , Tocchini‐Valentini, G. P. , Enright, A. J. , Norton, W. H. J. , Mandillo, S. , O'Carroll, D. , & Shkumatava, A. (2018). MicroRNA degradation by a conserved target RNA regulates animal behavior. Nature Structural & Molecular Biology, 25(3), 244–251. 10.1038/s41594-018-0032-x 29483647

[wrna1649-bib-0008] Chen, J. , Wassarman, K. M. , Feng, S. , Leon, K. , Feklistov, A. , Winkelman, J. T. , Li, Z. , Walz, T. , Campbell, E. A. , & Darst, S. A. (2017). 6S RNA mimics B‐form DNA to regulate *Escherichia coli* RNA polymerase. Molecular Cell, 68(2), 388–397.e6. 28988932 10.1016/j.molcel.2017.09.006 PMC5683422

[wrna1649-bib-0009] Cuturello, F. , Guido Tiana, G. , & Bussi, G. (2020). Assessing the accuracy of direct‐coupling analysis for RNA contact prediction. RNA, 26, 637–647. 32115426 10.1261/rna.074179.119 PMC7161351

[wrna1649-bib-0010] De Leonardis, E. , Lutz, B. , Ratz, S. , Cocco, S. , Monasson, R. , Schug, A. , & Weigt, M. (2015). Direct‐coupling analysis of nucleotide coevolution facilitates RNA secondary and tertiary structure prediction. Nucleic Acids Research, 43, 10444–10455. 26420827 10.1093/nar/gkv932 PMC4666395

[wrna1649-bib-0011] Deigan, K. E. , Li, T. W. , Mathews, D. H. , & Weeks, K. M. (2009). Accurate SHAPE‐directed RNA structure determination. Proceedings of the National Academy of Sciences of the United States of America, 106, 97–102. 19109441 10.1073/pnas.0806929106 PMC2629221

[wrna1649-bib-0012] del Val, C. , Rivas, E. , Torres‐Quesada, O. , Toro, N. , & Jiménez‐Zurdo, J. I. (2007). Identification of differentially expressed small non‐coding RNAs in the legume endosymbiont *Sinorhizobium meliloti* by comparative genomics. Molecular Microbiology, 66, 1080–1091. 17971083 10.1111/j.1365-2958.2007.05978.x PMC2780559

[wrna1649-bib-0013] Ding, Y. , Chan, C. Y. , & Lawrence, C. E. (2004). Sfold web server for statistical folding and rational design of nucleic acids. Nucleic Acids Research, 32, W135–W141. 15215366 10.1093/nar/gkh449 PMC441587

[wrna1649-bib-0014] Dirks, R. M. , & Pierce, N. A. (2003). A partition function algorithm for nucleic acid secondary structure including pseudoknots. Journal of Computational Chemistry, 24, 1664–1177. 12926009 10.1002/jcc.10296

[wrna1649-bib-0015] Do, C. B. , Woods, D. A. , & Batzoglou, S. (2006). CONTRAfold: RNA secondary structure prediction without physics‐based models. Bioinformatics, 22, e90–e98. 16873527 10.1093/bioinformatics/btl246

[wrna1649-bib-0016] Doshi, K. J. , Cannone, J. J. , Cobaugh, C. W. , & Gutell, R. R. (2004). Evaluation of the suitability of free‐energy minimization using nearest‐neighbor energy parameters for RNA secondary structure prediction. BMC Bioinformatics, 5, 105. 15296519 10.1186/1471-2105-5-105 PMC514602

[wrna1649-bib-0017] Dowell, R. D. , & Eddy, S. R. (2004). Evaluation of several lightweight stochastic context‐free grammars for RNA secondary structure prediction. BMC Bioinformatics, 5, 71. 15180907 10.1186/1471-2105-5-71 PMC442121

[wrna1649-bib-0018] Dunn, S. D. , Wahl, L. M. , & Gloor, G. B. (2007). Mutual information without the influence of phylogeny or entropy dramatically improves residue contact predictions. Bioinformatics, 24, 333–340. 18057019 10.1093/bioinformatics/btm604

[wrna1649-bib-0019] Durbin, R. , Eddy, S. R. , Krogh, A. , & Mitchison, G. J. (1998). Biological sequence analysis: Probabilistic models of proteins and nucleic acids. Cambridge, UK: Cambridge University Press.

[wrna1649-bib-0020] Dutheil, J. Y. (2012). Detecting coevolving positions in a molecule: Why and how to account for phylogeny. Briefings in Bioinformatics, 13, 228–243. 21949241 10.1093/bib/bbr048

[wrna1649-bib-0021] Eddy, S. R. (2014). Computational analysis of conserved RNA secondary structure in transcriptomes and genomes. Annual Review of Biophysics, 43, 433–456. 10.1146/annurev-biophys-051013-022950 PMC5541781 24895857

[wrna1649-bib-0022] Edgar, R. C. (2004). MUSCLE: A multiple sequence alignment method with reduced time and space complexity. BMC Bioinformatics, 5, 113. 15318951 10.1186/1471-2105-5-113 PMC517706

[wrna1649-bib-0023] Ekeberg, M. , Lövkvist, C. , Lan, Y. , Weigt, M. , & Aurell, E. (2013). Improved contact prediction in proteins: Using pseudolikelihoods to infer Potts models. Physical Review E, 87(1), 012707. 10.1103/PhysRevE.87.012707 23410359

[wrna1649-bib-0024] Fang, R. , Moss, W. N. , Rutenberg‐Schoenberg, M. , & Simon, M. D. (2015). Probing Xist RNA structure in cells using targeted structure‐seq. PLoS Genetics, 11(12), e1005668. 26646615 10.1371/journal.pgen.1005668 PMC4672913

[wrna1649-bib-0025] Flamm, C. , Fontana, W. , Hofacker, I. , & Schuster, P. (2000). RNA folding kinetics at elementary step resolution. RNA, 6, 325–338. 10744018 10.1017/s1355838200992161 PMC1369916

[wrna1649-bib-0026] Gautheret, D. , Konings, D. , & Gutell, R. (1994). A major family of motifs involving G.A mismatches in ribosomal RNA. Journal of Molecular Biology, 242, 1–8. 8078068 10.1006/jmbi.1994.1552

[wrna1649-bib-0027] Giegé, R. , Sissler, M. , & Florentz, C. (1998). Universal rules and idiosyncratic features in tRNA identity. Nucleic Acids Research, 26, 5017–5035. 9801296 10.1093/nar/26.22.5017 PMC147952

[wrna1649-bib-0028] Goudarzi, M. , Berg, K. , Pieper, L. M. , & Schier, A. F. (2019). Individual long non‐coding RNAs have no overt functions in zebrafish embryogenesis, viability and fertility. eLife, 8, e40815. 30620332 10.7554/eLife.40815 PMC6347452

[wrna1649-bib-0029] Gruber, A. R. , Bernhart, S. H. , & Lorenz, R. (2015). The ViennaRNA web services. In RNA bioinformatics (pp. 307–326). Springer. 10.1007/978-1-4939-2291-8_19 25577387

[wrna1649-bib-0030] Gruber, A. R. , Findeiß, S. , Washietl, S. , Hofacker, I. L. , & Stadler, P. F. (2010). RNAz 2.0: Improved noncoding RNA detection. Pacific Symposium on Biocomputing, 15, 69–79. 19908359

[wrna1649-bib-0031] Gutell, R. R. (2014). Ten lessons with Carl Woese about RNA and comparative analysis. RNA Biology, 11(3), 254–272. 24713659 10.4161/rna.28718

[wrna1649-bib-0032] Gutell, R. R. , Larsen, N. , & Woese, C. R. (1994). Lessons from an evolving rRNA: 16S and 23S rRNA structures from a comparative perspective. Microbiological Reviews, 58, 10–26. 8177168 10.1128/mr.58.1.10-26.1994 PMC372950

[wrna1649-bib-0033] Gutell, R. R. , Power, A. , Hertz, G. Z. , Putz, E. J. , & Stormo, G. D. (1992). Identifying constraints on the higher‐order structure of RNA: Continued development and application of comparative sequence analysis methods. Nucleic Acids Research, 20, 5785–5795. 1454539 10.1093/nar/20.21.5785 PMC334417

[wrna1649-bib-0034] Gutell, R. R. , Weiser, B. , Woese, C. R. , & Noller, H. F. (1985). Comparative anatomy of 16S‐like ribosomal RNA. Progress in Nucleic Acid Research and Molecular Biology, 32, 155–216. 3911275 10.1016/s0079-6603(08)60348-7

[wrna1649-bib-0035] Hamada, M. , Kiryu, H. , Sato, K. , Mituyama, T. , & Asai, K. (2009). Prediction of RNA secondary structure using generalized centroid estimators. Bioinformatics, 25, 465–473. 19095700 10.1093/bioinformatics/btn601

[wrna1649-bib-0036] Hawkes, E. J. , Hennelly, S. P. , Novikova, I. V. , Irwin, J. A. , Dean, C. , & Sanbonmatsu, K. Y. (2016). COOLAIR antisense RNAs form evolutionarily conserved elaborate secondary structures. Cell Reports, 16, 3087–3096. 27653675 10.1016/j.celrep.2016.08.045 PMC6827332

[wrna1649-bib-0037] Hofacker, I. L. , Fekete, M. , & Stadler, P. F. (2002). Secondary structure prediction for aligned RNA sequences. Journal of Molecular Biology, 319, 1059–1066. 12079347 10.1016/S0022-2836(02)00308-X

[wrna1649-bib-0038] Holley, R. W. , Apgar, J. , Everett, G. A. , Madison, J. T. , Marquisee, M. , Merrill, S. H. , Penswick, J. R. , & Zamir, A. (1965). Structure of a ribonucleic acid. Science, 14, 1462–1465. 10.1126/science.147.3664.1462 14263761

[wrna1649-bib-0039] Hunkler, H. J. , Hoepfner, J. , Huang, C.‐K. , Chatterjee, S. , Jara‐Avaca, M. , Gruh, I. , Bolesani, E. , Zweigerdt, R. , Thum, T. , & Bär, C. (2020). The long non‐coding RNA Cyrano is dispensable for pluripotency of murine and human pluripotent stem cells. Stem Cell Reports, 15(1), 13–21. 32531193 10.1016/j.stemcr.2020.05.011 PMC7363876

[wrna1649-bib-0040] Incarnato, D. , Morandi, E. , Anselmi, F. , Simon, L. M. , Basile, G. , & Oliviero, S. (2017). In vivo probing of nascent RNA structures reveals principles of cotranscriptional folding. Nucleic Acids Research, 45, 9716–9725. 28934475 10.1093/nar/gkx617 PMC5766169

[wrna1649-bib-0041] International Human Genome Sequencing Consortium . (2001). Initial sequencing and analysis of the human genome. Nature, 409, 860–921. 11237011 10.1038/35057062

[wrna1649-bib-0042] Jaynes, E. T. (1982). On the rationale of maximum‐entropy methods. Proceedings of the IEEE, 70, 939–952.

[wrna1649-bib-0043] Jones, A. N. , Pisignano, G. , Pavelitz, T. , White, J. , Kinisu, M. , Forino, N. , Dreycey1, A. , & Varani, G. (2020). An evolutionarily‐conserved RNA structure in the functional core of the lincRNA Cyrano. RNA, 26(9), 1234–1246. 10.1261/rna.076117.120 32457084 PMC7430676

[wrna1649-bib-0044] Jones, D. T. , Buchan, D. W. A. , Cozzetto, D. , & Pontil, M. (2012). PSICOV: Precise structural contact prediction using sparse inverse covariance estimation on large multiple sequence alignments. Bioinformatics, 28(2), 184–190. 22101153 10.1093/bioinformatics/btr638

[wrna1649-bib-0045] Kalvari, I. , Argasinska, J. , Quinones‐Olvera, N. , Nawrocki, E. P. , Rivas, E. , Eddy, S. R. , Bateman, A. , Finn, R. D. , & Petrov, A. I. (2018). Rfam 13.0: Shifting to a genome‐centric resource for non‐coding RNA families. Nucleic Acids Research, 46(D1), D335–D342. 29112718 10.1093/nar/gkx1038 PMC5753348

[wrna1649-bib-0046] Kamisetty, H. , Ovchinnikov, S. , & Baker, D. (2013). Assessing the utility of coevolution‐based residue–residue contact predictions in a sequence‐and structure‐rich era. Proceedings of the National Academy of Sciences of the United States of America, 110(39), 15674–15679. 24009338 10.1073/pnas.1314045110 PMC3785744

[wrna1649-bib-0047] Kleaveland, B. , Shi, C. Y. , Stefano, J. , & Bartel, D. P. (2018). A network of noncoding regulatory RNAs acts in the mammalian brain. Cell, 174(2), 350–362.e17. 29887379 10.1016/j.cell.2018.05.022 PMC6559361

[wrna1649-bib-0048] Layton, D. M. , & Bundschuh, R. (2005). A statistical analysis of RNA folding algorithms through thermodynamic parameter perturbation. Nucleic Acids Research, 33, 519–524. 15673712 10.1093/nar/gkh983 PMC548333

[wrna1649-bib-0049] Leontis, N. B. , & Westhof, E. (2001). Geometric nomenclature and classification of RNA base pairs. RNA, 7, 499–512. 11345429 10.1017/s1355838201002515 PMC1370104

[wrna1649-bib-0050] Lilley, D. M. J. (1995). Kinking of DNA and RNA by base bulges. Proceedings of the National Academy of Sciences of the United States of America, 92, 7140–7142. 7543675 10.1073/pnas.92.16.7140 PMC41294

[wrna1649-bib-0051] Lindgreen, S. , Gardner, P. P. , & Krogh, A. (2006). Measuring covariation in RNA alignments: Physical realism improves information measures. Bioinformatics, 22, 2988–2995. 17038338 10.1093/bioinformatics/btl514

[wrna1649-bib-0052] Liu, F. , Somarowthu, S. , & Pyle, A. M. (2017). Visualizing the secondary and tertiary architectural domains of lncRNA RepA. Nature Chemical Biology, 13, 282–289. 28068310 10.1038/nchembio.2272 PMC6432788

[wrna1649-bib-0053] Loughrey, D. , Watters, K. E. , Settle, A. H. , & Lucks, J. B. (2014). SHAPE‐Seq 2.0: Systematic optimization and extension of high‐throughput chemical probing of RNA secondary structure with next generation sequencing. Nucleic Acids Research, 42(21), e165–e165. 10.1093/nar/gku909 PMC4245970 25303992

[wrna1649-bib-0054] Lu, Z. J. , Gloor, J. W. , & Mathews, D. H. (2009). Improved RNA secondary structure prediction by maximizing expected pair accuracy. RNA, 15, 1805–1813. 19703939 10.1261/rna.1643609 PMC2743040

[wrna1649-bib-0055] Maenner, S. , Blaud, M. , Fouillen, L. , Savoye, A. , Marchand, V. , Dubois, A. , Sanglier‐Cianférani, S. , Dorsselaer, A. V. , Clerc, P. , Avner, P. , Visvikis, A. , & Branlant, C. (2010). 2‐D structure of the A region of Xist RNA and its implication for PRC2 association. PLoS Biology, 8, e1000276. 20052282 10.1371/journal.pbio.1000276 PMC2796953

[wrna1649-bib-0056] Manfredonia, I. , Nithin, C. , Ponce‐Salvatierra, A. , Ghosh, P. , Wirecki, T. K. , Marinus, T. , Ogando, N. S. , Snijder, E. , van Hemert, M. J. , Bujnicki, J. M. , & Incarnato, D. (2020). Genome‐wide mapping of SARS‐CoV‐2 RNA structures identifies therapeutically‐relevant elements. Nucleic Acids Research, 48(22), 12436–12452. 33166999 10.1093/nar/gkaa1053 PMC7736786

[wrna1649-bib-0057] Marks, D. S. , Colwell, L. J. , Sheridan, R. , Hopf, T. A. , Pagnani, A. , Zecchina, R. , & Sander, C. (2011). Protein 3D structure computed from evolutionary sequence variation. PLoS One, 6(12), e28766. 22163331 10.1371/journal.pone.0028766 PMC3233603

[wrna1649-bib-0058] Michel, F. , Costa, M. , Massire, C. , & Westhof, E. (2000). Modeling RNA tertiary structure from patterns of sequence variation. Methods in Enzymology, 317, 491–510. 10829297 10.1016/s0076-6879(00)17031-4

[wrna1649-bib-0059] Michel, F. , Jacquier, A. , & Dujon, B. (1982). Comparison of fungal mitochondrial introns reveals extensive homologies in RNA secondary structure. Biochimie, 64, 867–881. 6817818 10.1016/s0300-9084(82)80349-0

[wrna1649-bib-0060] Mironov, A. S. , & Lebedev, V. F. (1993). A kinetic model of RNA folding. Boiosystems, 30, 49–56. 10.1016/0303-2647(93)90061-g 7690611

[wrna1649-bib-0061] Montange, R. , & Batey, R. T. (2006). Structure of the S‐adenosylmethionine riboswitch regulatory mRNA element. Nature, 441, 1172–1175. 16810258 10.1038/nature04819

[wrna1649-bib-0062] Morcos, F. , Pagnani, A. , Lunt, B. , Bertolino, A. , Marks, D. S. , Sander, C. , Zecchina, R. , Onuchic, J. N. , Hwa, T. , & Weigt, M. (2011). Direct‐coupling analysis of residue coevolution captures native contacts across many protein families. Proceedings of the National Academy of Sciences of the United States of America, 108, E1293–E1301. 22106262 10.1073/pnas.1111471108 PMC3241805

[wrna1649-bib-0063] Mustoe, A. M. , Lama, N. N. , Irving, P. S. , Olson, S. W. , & Weeks, K. M. (2019). RNA base‐pairing complexity in living cells visualized by correlated chemical probing. Proceedings of the National Academy of Sciences of the United States of America, 116(49), 24574–24582. 31744869 10.1073/pnas.1905491116 PMC6900531

[wrna1649-bib-0064] Nawrocki, E. P. , & Eddy, S. R. (2013). Infernal 1.1: 100‐fold faster RNA homology searches. Bioinformatics, 29, 2933–2935. 24008419 10.1093/bioinformatics/btt509 PMC3810854

[wrna1649-bib-0065] Noller, H. F. , Kop, J. , Wheaton, V. , Brosius, J. , Gutell, R. R. , Kopylov, A. M. , Dohme, F. , Herr, W. , Stahl, D. A. , Gupta, R. , & Woese, C. R. (1981). Secondary structure model for 23S ribosomal RNA. Nucleic Acids Research, 9, 6167–6189. 7031608 10.1093/nar/9.22.6167 PMC327592

[wrna1649-bib-0066] Novikova, I. V. , Hennelly, S. P. , & Sanbonmatsu, K. Y. (2012). Structural architecture of the human long non‐coding RNA, steroid receptor RNA activator. Nucleic Acids Research, 40, 5034–5051. 22362738 10.1093/nar/gks071 PMC3367176

[wrna1649-bib-0067] Nussinov, R. , Pieczenik, G. , Griggs, J. R. , & Kleitman, D. J. (1978). Algorithms for loop matchings. SIAM Journal on Applied Mathematics, 35, 68–82.

[wrna1649-bib-0068] Pace, N. R. , Smith, D. K. , Olsen, G. J. , & James, B. D. (1989). Phylogenetic comparative analysis and the secondary structure of ribonuclease P RNA – A review. Gene, 82, 65–75. 2479592 10.1016/0378-1119(89)90031-0

[wrna1649-bib-0069] Parker, B. J. , Moltke, I. , Roth, A. , Washietl, S. , Wen, J. , Kellis, M. , Breaker, R. , & Pedersen, J. S. (2011). New families of human regulatory RNA structures identified by comparative analysis of vertebrate genomes. Genome Research, 21, 1929–1943. 21994249 10.1101/gr.112516.110 PMC3205577

[wrna1649-bib-0070] Pedersen, J. S. , Bejerano, G. , Siepel, A. , Rosenbloom, K. , Lindblad‐Toh, K. , Lander, E. S. , Kent, J. , Miller, W. , & Haussler, D. (2006). Identification and classification of conserved RNA secondary structures in the human genome. PLoS Computational Biology, 2, e33. 16628248 10.1371/journal.pcbi.0020033 PMC1440920

[wrna1649-bib-0071] Perriman, R. J. , & Ares, M. (2007). Rearrangement of competing U2 RNA helices within the spliceosome promotes multiple steps in splicing. Genes & Development, 21(7), 811–820. 17403781 10.1101/gad.1524307 PMC1838532

[wrna1649-bib-0072] Perriman, R. J. , & Ares, M. (2010). Invariant U2 snRNA nucleotides form a stem loop to recognize the intron early in splicing. Molecular Cell, 38(3), 416–427. 20471947 10.1016/j.molcel.2010.02.036 PMC2872779

[wrna1649-bib-0073] Potts, R. B. (1952). Some generalized order–disorder transformations. Mathematical Proceedings of the Cambridge Philosophical Society, 48, 106–109.

[wrna1649-bib-0074] Ramrath, D. , Yamamoto, H. , Rother, K. , Wittek, D. , Pech, M. , Mielke, T. , Justus Loerke, J. , Scheerer, P. , Ivanov, P. , Teraoka, Y. , Shpanchenko, O. , Nierhaus, K. H. , & Spahn, C. M. T. (2012). The complex of tmRNA–SmpB and EF‐G on translocating ribosomes. Nature, 485, 526–529. 22622583 10.1038/nature11006

[wrna1649-bib-0075] Rangan, R. , Zheludev, I. N. , & Das, R. (2020). RNA genome conservation and secondary structure in SARS‐CoV‐2 and SARS‐related viruses: A first look. RNA, 26, 937–959. 32398273 10.1261/rna.076141.120 PMC7373990

[wrna1649-bib-0076] Reuter, J. S. , & Mathews, D. H. (2010). RNAstructure: Software for RNA secondary structure prediction and analysis. BMC Bioinformatics, 11, 10. 20230624 10.1186/1471-2105-11-129 PMC2984261

[wrna1649-bib-0077] Ritz, J. , Martin, J. S. , & Laederach, A. (2013). Evolutionary evidence for alternative structure in RNA sequence co‐variation. PLoS Computational Biology, 9, e1003152. 23935473 10.1371/journal.pcbi.1003152 PMC3723493

[wrna1649-bib-0078] Rivas, E. (2013). The four ingredients of single‐sequence RNA secondary structure prediction: A unifying perspective. RNA Biology, 10, 1185–1196. 23695796 10.4161/rna.24971 PMC3849167

[wrna1649-bib-0079] Rivas, E. (2020). RNA structure prediction using positive and negative evolutionary information. PLoS Computational Biology, 16(10), e1008387. 33125376 10.1371/journal.pcbi.1008387 PMC7657543

[wrna1649-bib-0080] Rivas, E. , Clements, J. , & Eddy, S. R. (2017). A statistical test for conserved RNA structure shows lack of evidence for structure in lncRNAs. Nature Methods, 14, 45–48. 27819659 10.1038/nmeth.4066 PMC5554622

[wrna1649-bib-0081] Rivas, E. , Clements, J. , & Eddy, S. R. (2020). Estimating the power of sequence covariation for detecting conserved RNA structure. Bioinformatics, 36, 3072–3076. 32031582 10.1093/bioinformatics/btaa080 PMC7214042

[wrna1649-bib-0082] Rivas, E. , & Eddy, S. R. (1999). A dynamic programming algorithm for RNA structure prediction including pseudoknots. Journal of Molecular Biology, 285, 2053–2068. 9925784 10.1006/jmbi.1998.2436

[wrna1649-bib-0083] Rivas, E. , & Eddy, S. R. (2000). Secondary structure alone is generally not statistically significant for the detection of noncoding RNAs. Bioinformatics, 6, 583–605. 10.1093/bioinformatics/16.7.583 11038329

[wrna1649-bib-0084] Rivas, E. , & Eddy, S. R. (2001). Noncoding RNA gene detection using comparative sequence analysis. BMC Bioinformatics, 2, 8. 11801179 10.1186/1471-2105-2-8 PMC64605

[wrna1649-bib-0085] Rivas, E. , & Eddy, S. R. (2018). Response to Tavares et al., “covariation analysis with improved parameters reveals conservation in lncRNA structures”. bioRxiv. 10.1101/2020.02.18.955047 PMC6515926 30890332

[wrna1649-bib-0086] Rivas, E. , Klein, R. J. , Jones, T. A. , & Eddy, S. R. (2001). Computational identification of noncoding RNAs in *E. coli* by comparative genomics. Current Biology, 11, 1369–1373. 11553332 10.1016/s0960-9822(01)00401-8

[wrna1649-bib-0087] Rivas, E. , Lang, R. , & Eddy, S. R. (2012). A range of complex probabilistic models for RNA secondary structure prediction that include the nearest neighbor model and more. RNA, 18, 193–212. 22194308 10.1261/rna.030049.111 PMC3264907

[wrna1649-bib-0088] Shannon, C. (1948). A note on the concept of entropy. The Bell System Technical Journal, 27, 379–423.

[wrna1649-bib-0089] Somarowthu, S. , Legiewicz, M. , Chillón, I. , Marcia, M. , Liu, F. , & Pyle, A. M. (2015). HOTAIR forms an intricate and modular secondary structure. Molecular Cell, 58, 353–361. 25866246 10.1016/j.molcel.2015.03.006 PMC4406478

[wrna1649-bib-0090] Sørensen, P. D. , Lomholt, B. , Frederiksen, S. , & Tommerup, N. (1991). Fine mapping of human 5S rRNA genes to chromosome 1q42.11–q42.13. Cytogenetics and Cell Genetics, 57, 26–29. 1855389 10.1159/000133107

[wrna1649-bib-0091] Sükösd, Z. , Swenson, M. S. , Kjems, J. , & Heitsch, C. E. (2013). Evaluating the accuracy of SHAPE‐directed RNA secondary structure predictions. Nucleic Acids Research, 41, 2807–2816. 23325843 10.1093/nar/gks1283 PMC3597644

[wrna1649-bib-0092] Swenson, M. S. , Anderson, J. , Ash, A. , Gaurav, P. , Sükösd, Z. , Bader, D. A. , Harvey, S. C. , & Heitsch, C. E. (2012). GTfold: Enabling parallel RNA secondary structure prediction on multi‐core desktops. BMC Research Notes, 5, 341. 22747589 10.1186/1756-0500-5-341 PMC3748833

[wrna1649-bib-0093] Tavares, R. C. , Pyle, A. M. , & Somarowthu, S. (2019). Phylogenetic analysis with improved parameters reveals conservation in lncRNA structures. Journal of Molecular Biology, 431(8), 1592–1603. 30890332 10.1016/j.jmb.2019.03.012 PMC6515926

[wrna1649-bib-0094] Torarinsson, E. , Sawera, M. , Havgaard, J. H. , Fredholm, M. , & Gorodkin, J. (2006). Thousands of corresponding human and mouse genomic regions unalignable in primary sequence contain common RNA structure. Genome Research, 16, 885–889. 16751343 10.1101/gr.5226606 PMC1484455

[wrna1649-bib-0095] Torarinsson, E. , Yao, Z. , Wiklund, E. D. , Bramsen, J. B. , Hansen, C. , Kjems, J. , Tommerup, N. , Ruzzo, W. L. , & Gorodkin, J. (2008). Comparative genomics beyond sequence‐based alignments: RNA structures in the ENCODE regions. Genome Research, 18, 242–251. 18096747 10.1101/gr.6887408 PMC2203622

[wrna1649-bib-0096] Ulitsky, I. , Shkumatava, A. , Jan, C. H. , Sive, H. , & Bartel, D. P. (2011). Conserved function of lincRNAs in vertebrate embryonic development despite rapid sequence evolution. Cell, 147, 1537–1550. 22196729 10.1016/j.cell.2011.11.055 PMC3376356

[wrna1649-bib-0097] Washietl, S. , Hofacker, I. L. , Lukasser, M. , Hüttenhofer, A. , & Stadler, P. F. (2005). Mapping of conserved RNA secondary structures predicts thousands of functional noncoding RNAs in the human genome. Nature Biotechnology, 23, 1383–1390. 10.1038/nbt1144 16273071

[wrna1649-bib-0098] Washietl, S. , Hofacker, I. L. , Stadler, P. F. , & Kellis, M. (2012). RNA folding with soft constraints: Reconciliation of probing data and thermodynamic secondary structure prediction. Nucleic Acids Research, 40, 4261–4272. 22287623 10.1093/nar/gks009 PMC3378861

[wrna1649-bib-0099] Washietl, S. , Pedersen, J. S. , Korbel, J. O. , Stocsits, C. , Gruber, A. R. , Hackermüller, J. , Hertel, J. , Lindemeyer, M. , Reiche, K. , Tanzer, A. , Ucla, C. , Wyss, C. , Antonarakis, S. E. , Denoeud, F. , Lagarde, J. , Drenkow, J. , Kapranov, P. , Gingeras, T. R. , Guigó, R. , … Stadler, P. F. (2007). Structured RNAs in the ENCODE selected regions of the human genome. Genome Research, 17, 852–864. 17568003 10.1101/gr.5650707 PMC1891344

[wrna1649-bib-0100] Washietl, S. , Wen, J. , Kellis, M. , Breaker, R. , & Pedersen, J. S. (2011). New families of human regulatory RNA structures identified by comparative analysis of vertebrate genomes. Genome Research, 21, 1929–1943. 21994249 10.1101/gr.112516.110 PMC3205577

[wrna1649-bib-0101] Watts, J. M. , Dang, K. K. , Gorelick, R. J. , Leonard, C. W. , Bess, J. W., Jr. , Swanstrom, R. , Burch, C. L. , & Weeks, K. M. (2009). Architecture and secondary structure of an entire HIV‐1 RNA genome. Nature, 460, 711–716. 19661910 10.1038/nature08237 PMC2724670

[wrna1649-bib-0102] Weigt, M. , White, R. A. , Szurmant, H. , Hoch, J. A. , & Hwa, T. (2009). Identification of direct residue contacts in protein–protein interaction by message passing. Proceedings of the National Academy of Sciences of the United States of America, 106, 67–72. 19116270 10.1073/pnas.0805923106 PMC2629192

[wrna1649-bib-0103] Weinberg, Z. , & Breaker, R. R. (2011). R2R – Software to speed the depiction of aesthetic consensus RNA secondary structures. BMC Bioinformatics, 12, 3. 21205310 10.1186/1471-2105-12-3 PMC3023696

[wrna1649-bib-0104] Weinreb, C. , Riesselman, A. J. , Ingraham, J. B. , Gross, T. , Sander, C. , & Marks, D. S. (2016). 3D RNA and functional interactions from evolutionary couplings. Cell, 165, 963–975. 27087444 10.1016/j.cell.2016.03.030 PMC5024353

[wrna1649-bib-0105] Williams, K. P. , & Bartel, D. P. (1996). Phylogenetic analysis of tmRNA secondary structure. RNA, 2, 1306–1310. 8972778 PMC1369456

[wrna1649-bib-0106] Wolfinger, M. T. , Svrcek‐Seiler, W. A. , Flamm, C. , & Hofacker, I. L. (2004). Exact folding dynamics of RNA secondary structures. Journal of Physics A: Mathematical and General, 37, 4731–4474.

[wrna1649-bib-0107] Woolf, B. (1957). The log likelihood ratio test (the G‐test). Annals of Human Genetics, 21, 397–409. 13435648 10.1111/j.1469-1809.1972.tb00293.x

[wrna1649-bib-0108] Yang, H. , Jossinet, F. , Leontis, N. , Chen, L. , Westbrook, J. , Berman, H. M. , & Westhof, E. (2003). Tools for the automatic identification and classification of RNA base pairs. Nucleic Acids Research, 31(13), 3450–3460. 12824344 10.1093/nar/gkg529 PMC168936

[wrna1649-bib-0109] Yao, Z. , Weinberg, Z. , & Ruzzo, W. L. (2006). CMfinder – A covariance model based RNA motif finding algorithm. Bioinformatics, 22, 445–452. 16357030 10.1093/bioinformatics/btk008

[wrna1649-bib-0110] Yarus, M. (1982). Translational efficiency of transfer RNA's: Uses of an extended anticodon. Science, 218, 646–652. 6753149 10.1126/science.6753149

[wrna1649-bib-0111] Yeang, C.‐H. , & Haussler, D. (2007). Detecting coevolution in and among protein domains. PLOS Computational Biology, 3(11), e211. 17983264 10.1371/journal.pcbi.0030211 PMC2098842

[wrna1649-bib-0112] Zarringhalam, K. , Meyer, M. M. , Dotu, I. , Chuang, J. H. , & Clote, P. (2012). Integrating chemical footprinting data into RNA secondary structure prediction. PLoS One, 7, e45160. 23091593 10.1371/journal.pone.0045160 PMC3473038

[wrna1649-bib-0113] Zhang, Q. , Kim, N.‐K. , Peterson, R. D. , Wang, Z. , & Feigon, J. (2010). Structurally conserved five nucleotide bulge determines the overall topology of the core domain of human telomerase RNA. Proceedings of the National Academy of Sciences of the United States of America, 107(44), 18761–18768. 20966348 10.1073/pnas.1013269107 PMC2973926

[wrna1649-bib-0114] Zhu, J. Y. A. , & Meyer, I. M. (2015). Four RNA families with functional transient structures. RNA Biology, 12, 5–20. 25751035 10.1080/15476286.2015.1008373 PMC4615214

[wrna1649-bib-0115] Zuker, M. (1989). On finding all suboptimal foldings of an RNA molecule. Science, 244, 48–52. 2468181 10.1126/science.2468181

[wrna1649-bib-0116] Zuker, M. , & Sankoff, S. (1984). RNA secondary structures and their prediction. Bulletin of Mathematical Biology, 46, 591–621.

