# Supplementary material for: Evolutionary conservation of RNA sequence and structure
Source: Wiley Interdiscip Rev RNA. 2021 Mar 22;12(5):e1649. doi: 10.1002/wrna.1649 (PMC8250186; doi:10.1002/wrna.1649)
Supplement: Supplementary file 1 — TABLE S1 Detection of RNA base pairs by different covariation measures. The Rfam seed alignments come from Rfam v 14.2. The Rfam full alignments are those provided in Ref. Weinreb et al., 2016. The structural annotation was derived from the PDB files using the program RNAview (Yang et al., 2003), obtained by the program R‐scape using option: –pdb. For a given RNA family, both the Seed and Full alignments have annotated the same structure. The structure includes both the Watson‐Crick (WC) base pairs as well as the non‐canonical base pairs (nonWC). G‐test scores are calculated using the program R‐scape (using option: –naive), and they include the average product correction (APC) (Dunn et al., 2007). Scores from DCA couplings trained using Boltzmann Learning optimization (BL‐DCA) are calculated using the code provided with Ref. Cuturello et al., 2020. ECs scores trained using pseudo‐maximum likelihood optimization come directly from Ref. Weinreb et al., 2016. Running times for ECs are unavailable. PPV stands for positive predictive value, and it is the fraction of the detected basepairs that are true base pairs. [file WRNA-12-0-s002.pdf]

Table S1. **Detection of RNA base pairs by different covariation measures.** The Rfam seed alignments come from Rfam v 14.2. The Rfam full alignments are those provided in Ref. (Weinreb et al., 2016). The structural annotation was derived from the PDB files using the program RNAview (Yang et al., 2003), obtained by the program R-scape using option: `--pdb`. For a given RNA family, both the Seed and Full alignments have annotated the same structure. The structure includes both the Watson-Crick (WC) base pairs as well as the non-canonical base pairs (nonWC). G-test scores are calculated using the program R-scape (using option: `--naive`), and they include the average product correction (APC) (Dunn et al., 2007). Scores from DCA couplings trained using Boltzmann Learning optimization (BL-DCA) are calculated using the code provided with Ref. (Cuturello et al., 2020). ECs scores trained using pseudo-maximum likelihood optimization come directly from Ref. (Weinreb et al., 2016). Running times for ECs are unavailable. PPV stands for positive predictive value, and it is the fraction of the detected basepairs that are true base pairs.

		Base Pairs	Alignment			Covariation	Detected at 50% PPV		Time
RNA	PDB	WC/nonWC		#seq	avg len	Method	WC	nonWC	(min)
5.8S rRNA RF00002	1KQS	25/0	Seed	61	152	G-test	52%(13/25)	0% (0/0)	0.01
						BL-DCA	44%(11/25)	0% (0/0)	67.60
			Full	375,612	153	G-test	60%(15/25)	0% (0/0)	33.53
						BL-DCA	24%(6/25)	0% (0/0)	38.93
						ECs	56%(14/25)	0% (0/0)	–
tRNA RF00005	1FIR	23/8	Seed	954	73	G-test	96%(22/23)	88%(7/8)	0.06
						BL-DCA	100%(23/23)	100%(8/8)	24.65
			Full	298,470	70	G-test	91%(21/23)	50%(4/8)	27.19
						BL-DCA	87%(20/23)	38%(3/8)	6.51
						ECs	100%(23/23)	100%(8/8)	–
RNase P RNA bacterial A RF00010	3Q1Q	128/21	Seed	458	380	G-test	74%(95/128)	0%(0/21)	0.11
						BL-DCA	75%(96/128)	5%(1/21)	1999.27
			Full	6,397	330	G-test	82%(105/128)	0%(0/21)	0.95
						BL-DCA	81%(104/128)	5%(1/21)	238.05
						ECs	83%(106/128)	14%(3/21)	–
SRP Metazoan RF00017	1RY1	126/13	Seed	91	293	G-test	40%(50/126)	0%(0/13)	0.01
						BL-DCA	0%(0/126)	0%(0/13)	301.89
			Full	22,685	282	G-test	71%(89/126)	1%(1/13)	3.67
						BL-DCA	0%(0/126)	0%(0/13)	185.03
						ECs	40%(50/126)	0%(0/13)	–
transfer-messenger RNA RF00023	3IYQ	128/16	Seed	477	357	G-test	80%(102/128)	0%(0/16)	0.10
						BL-DCA	81%(104/128)	6%(1/16)	1614.60
			Full	4,516	123	G-test	50%(64/128)	0%(0/16)	0.89
						BL-DCA	50%(64/128)	0%(0/16)	279.37
						ECs	51%(65/128)	0%(0/16)	–
FMN riboswitch RF00050	3F2X	34/15	Seed	144	136	G-test	44%(15/34)	13%(2/15)	0.01
						BL-DCA	50%(17/34)	13%(2/15)	78.68
			Full	4,516	123	G-test	59%(20/34)	13%(2/15)	0.16
						BL-DCA	32%(11/34)	7%(1/15)	32.57
						ECs	79%(27/34)	20%(3/15)	–

Continues on next page

Table S1 – *Continues from previous page*

RNA	PDB	Base Pairs WC/nonWC	Alignment	#seq	avg len	Covariation Method	Detected at 50% PPV WC	nonWC	Time (min)
TPP riboswitch RF00059	2GDI	33/9	Seed	109	107	G-test	76%(25/33)	0%(0/9)	0.01
						BL-DCA	70%(23/33)	0%(0/9)	69.84
			Full	11,197	97	G-test	82%(27/33)	0%(0/9)	0.42
						BL-DCA	79%(26/33)	0%(1/9)	14.43
						ECs	61%(20/33)	0%(0/9)	–
SAM-I riboswitch RF00162	4KQY	37/7	Seed	433	111	G-test	78%(29/37)	14%(1/7)	0.03
						BL-DCA	78%(29/37)	14%(1/7)	86.84
			Full	4,757	102	G-test	57%(21/37)	14%(1/7)	0.15
						BL-DCA	57%(21/37)	14%(1/7)	15.39
						ECs	70%(26/37)	14%(1/7)	–
Purine riboswitch RF00167	1Y26	26/7	Seed	133	101	G-test	81%(21/26)	0%(0/7)	0.01
						BL-DCA	65%(17/26)	0%(0/7)	16.55
			Full	2,427	100	G-test	81%(21/26)	0%(0/7)	0.06
						BL-DCA	81%(21/26)	0%(0/7)	13.91
						ECs	81%(21/26)	14%(1/7)	–
bacterial small SRP RF00169	2XXA	35/2	Seed	261	86	G-test	86%(30/35)	50%(1/2)	0.01
						BL-DCA	89%(31/35)	50%(1/2)	21.80
			Full	5,622	96	G-test	83%(29/35)	100%(2/2)	0.17
						BL-DCA	86%(30/35)	100%(2/2)	12.36
						ECs	91%(32/35)	100%(2/2)	–
Cobalamin riboswitch RF00174	4GXY	54/14	Seed	240	203	G-test	83%(45/54)	14%(2/14)	0.06
						BL-DCA	78%(42/54)	21%(3/14)	895.64
			Full	9,056	167	G-test	87%(47/54)	14%(2/14)	0.75
						BL-DCA	87%(47/54)	21%(3/14)	50.12
						ECs	91%(49/54)	21%(3/14)	–
GlmS ribozyme RF00234	2H0Z	48/11	Seed	18	182	G-test	44%(21/48)	0%(0/11)	0.00
						BL-DCA	0%(0/48)	0%(0/11)	257.10
			Full	842	151	G-test	71%(34/48)	0%(0/11)	0.04
						BL-DCA	65%(31/48)	9%(1/11)	35.31
						ECs	50%(24/48)	9%(1/11)	–
YkoK leader RF00380	3PDR	60/15	Seed	157	169	G-test	52%(31/60)	13%(2/15)	0.02
						BL-DCA	55%(33/60)	7%(1/15)	345.55
			Full	1,493	165	G-test	53%(32/60)	7%(1/15)	0.05
						BL-DCA	43%(26/60)	0%(0/15)	40.63
						ECs	68%(41/60)	13%(2/15)	–
Glycine riboswitch RF00504	3P49	26/2	Seed	44	101	G-test	58%(15/26)	0%(0/2)	0.00
						BL-DCA	54%(14/26)	0%(0/2)	211.06
			Full	6,875	80	G-test	65%(17/26)	0%(0/2)	10.7
						BL-DCA	65%(17/26)	0%(0/2)	13.49
						ECs	58%(15/26)	0%(0/2)	–
Fluoride riboswitch RF01734	4ENC	14/4	Seed	287	70	G-test	79%(11/14)	0%(0/4)	0.01
						BL-DCA	71%(10/14)	0%(0/4)	216.42
			Full	1,267	62	G-test	86%(12/14)	25%(1/4)	0.03
						BL-DCA	79%(11/14)	0%(0/4)	4.80
						ECs	93%(13/14)	25%(1/4)	–
Cyclic di-GMP-II riboswitch RF01786	3Q3Z	33/7	Seed	54	85	G-test	73%(24/33)	0%(0/7)	0.00
						BL-DCA	76%(25/33)	0%(0/7)	68.45
			Full	237	84	G-test	73%(24/33)	14%(1/7)	0.01
						BL-DCA	64%(21/33)	0%(0/7)	9.00
						ECs	70%(23/33)	14%(1/7)	–

Continues on next page

Table S1 – *Continues from previous page*

RNA	PDB	Base Pairs WC/nonWC	Alignment #seq	Alignment avg len	Covariation Method	Detected at 50% PPV WC	Detected at 50% PPV nonWC	Time (min)
THF riboswitch RF01831	4LVV	42/10	Seed 97	100	G-test	69%(29/42)	0%(0/10)	0.01
					BL-DCA	71%(30/42)	10%(1/10)	138.56
			Full 598	91	G-test	64%(27/42)	0%(0/10)	0.02
					BL-DCA	57%(24/42)	0%(0/10)	12.75
					ECs	55%(23/42)	10%(1/10)	–
Selenocysteine transfer RNA RF01852	3H2L	32/2	Seed 109	91	G-test	81%(26/32)	50%(1/2)	0.01
					BL-DCA	81%(26/32)	50%(1/2)	69.82
			Full 1,959	89	G-test	81%(26/32)	50%(1/2)	0.05
					BL-DCA	81%(26/32)	50%(1/2)	14.32
					ECs	69%(22/32)	50%(1/2)	–
Group II catalytic intron D1-D4-3 RF02001	3BWP	49/0	Seed 406	173	G-test	73%(36/49)	0%(0/0)	0.04
					BL-DCA	76%(37/49)	0%(0/0)	709.75
			Full 2,432	168	G-test	69%(34/49)	0%(0/0)	0.12
					BL-DCA	67%(33/49)	0%(0/0)	42.99
					ECs	80%(39/49)	0%(0/0)	–
