# Supplementary figures and images for: Evolutionary conservation of RNA sequence and structure
Source: Wiley Interdiscip Rev RNA. 2021 Mar 22;12(5):e1649. doi: 10.1002/wrna.1649 (PMC8250186; doi:10.1002/wrna.1649)

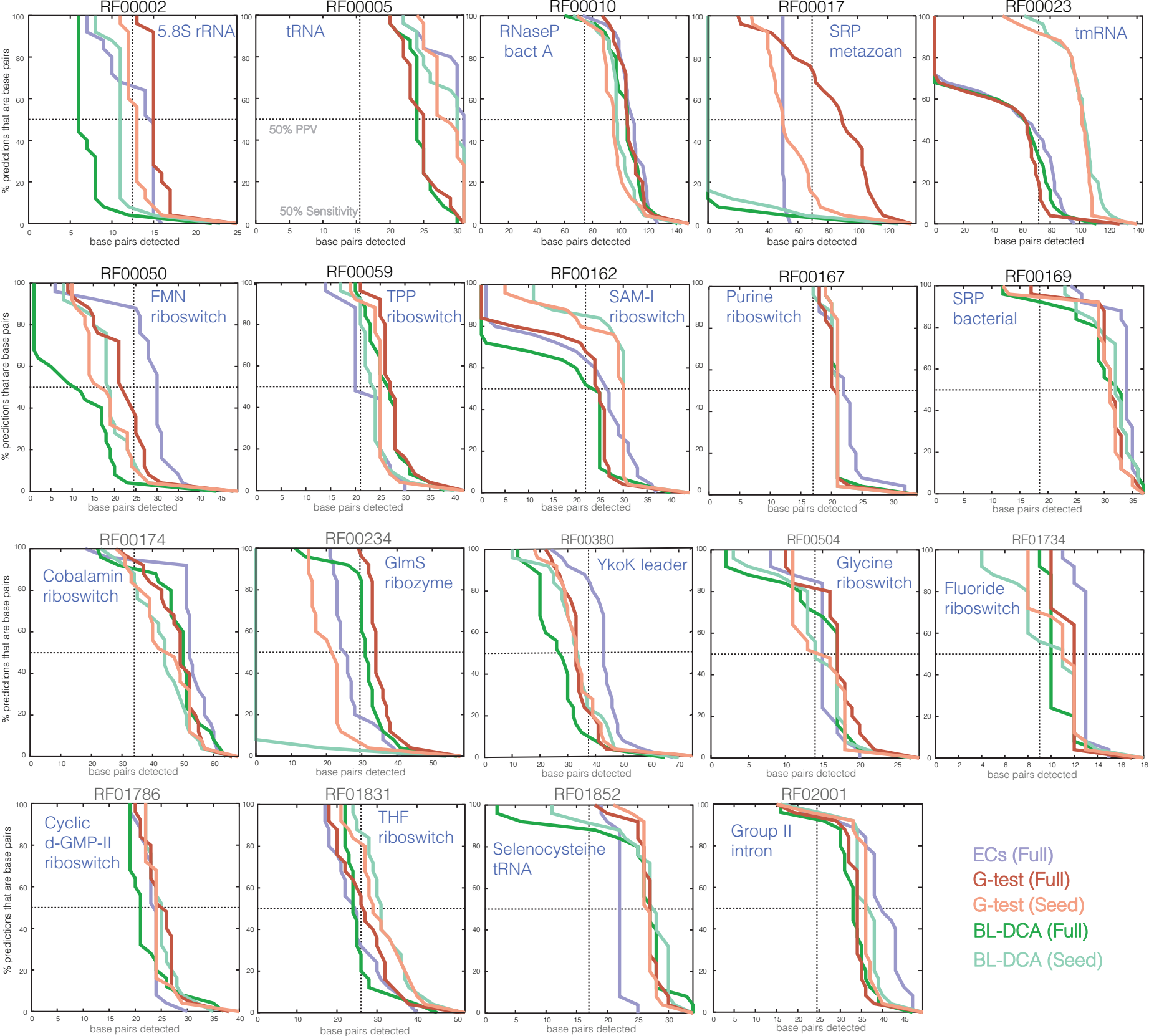

Supplement: Supplementary file 2 — FIGURE S1 Comparison of different measures of covariation on 19 structural RNAs with experimentally determined structures. For each structural RNA, we compare the performance of three different methods, on two different alignments. One of the methods is G‐test, a simple covariation measure calculated directly from the alignment (generated by the software R‐scape using option ––naive). The other two are DCA methods: EC_RNA (Weinreb et al., 2016) uses pseudo maximum likelihood, and BL‐DCA (Cuturello et al., 2020) uses a Boltzmann Machine to optimize the DCA coupling. All covariation methods use an APC correction (Dunn et al., 2007). The Seed alignments are from Rfam v14.2. The Full alignments are those used in (Weinreb et al., 2016). The base pairs has been inferred from a crystal structure (details given in Supplemental Table S1). The dashed lines correspond to 50% PPV (horizontal) and 50% sensitivity (vertical). [file WRNA-12-0-s001.pdf]
